# Low-dose radiation exaggerates HFD-induced metabolic dysfunction by gut microbiota through PA-PYCR1 axis

**DOI:** 10.1038/s42003-022-03929-1

**Published:** 2022-09-10

**Authors:** Zhao Ju, Peiyu Guo, Jing Xiang, Ridan Lei, Guofeng Ren, Meiling Zhou, Xiandan Yang, Pingkun Zhou, Ruixue Huang

**Affiliations:** 1 Department of Occupational and Environmental Health, Xiangya School of Public Health, Central South University, Changsha, Hunan Province 410078 China; 2 Department of Radiation Biology, Beijing Key Laboratory for Radiobiology, Beijing Institute of Radiation Medicine, AMMS, Beijing, China; 3 Department of Nutrition and Food Hygiene, Xiangya School of Public Health, Central South University, Changsha, Hunan Province 410078 China

**Keywords:** Chemical ecology, Non-alcoholic fatty liver disease

## Abstract

Co-exposure of High-fat-diet (HFD) behavior and environmental low-dose radiation (LDR) is common among majority occupational workers, but the synergism of this co-exposure in metabolic health is poorly understood. This study aimed to investigate the impact of gut microbiota and its metabolites on the regulation of HFD accompanied by LDR-associated with metabolic dysfunction and insulin resistance. Here, we reported that Parasutterella was markedly elevated in the gut microbiota of mice in co-exposure of HFD and LDR, accompanied by increased pyrrolidinecarboxylic acid (PA) level in both intestine and plasma. Transplantation of fecal microbiota from mice with co-exposure HFD and LDR with metabolic dysfunction resulted in increased disruption of metabolic dysfunction, insulin resistance and increased PYCR1 (Pyrroline-5-carboxylate reductase 1) expression. Mechanistically, intestinal barrier was damaged more serious in mice with co-exposure of HFD and LDR, leading high PA level in plasma, activating PYCR1 expression to inhibit insulin *Akt/mTOR* (AKT kinase-transforming protein/Serine threonine-protein kinase) signaling pathway to aggravate HFD-induced metabolic impairments. This study suggests a new avenue for interventions against western diet companied with low dose radiation exposure-driven metabolic impairments.

## Abstract (web summary)

Characterisation of gut microbiota shows that low-dose radiation enhances the metabolic defects induced by high-fat diet in mice, in a manner involving increased levels of pyrrolidinecarboxylic acid.

## Introduction

During the past three decades, the prevalence of obesity and its associated comorbidities, such as diabetes, have dramatically increased, becoming the most common chronic illnesses worldwide^1^ and resulting in almost 3 million deaths annually^2^. In addition, these common metabolic disorders pose major socioeconomic burdens^3^. The aetiologies of metabolic disorders have been reported to be multifactorial, includinghigh-fat diet (HFD) consumption^4,5^ and environmental factors such as air pollution^6^ and ionizing radiation^7,8^. Therefore, there is an urgent need to further understand how these multiple factors affect the development and progression of metabolic dysfunction.

Recently, the crucial roles of the gut microbiota in regulating obesity, diabetes, and insulin resistance through the generation of critical microbial metabolites have been revealed in vivo and in population-based studies^9,10^. Increasing evidence has indicated that the gut microbiota, along with its microbial metabolites, contributes to the close connections between environmental factors and host physiological and pathological status^11–13^. Gut microbiota homeostasis may enable the gut to play a role in mediating host metabolic health by regulating the intestinal barrier, gene signal transduction and energy homeostasis^14,15^. Notably, inter-individual variations in gut microbiota composition and various microbial metabolites, such as lipopolysaccharide (LPS), short-chain fatty acids(SCFAs) and amino acids^16,17^, are typically driven by an individual’s dietary habits and exposure to environmental factors. The gut microbiota and microbial metabolites also show positive or negative associations with host metabolic function in various contexts. For instance, LPS has a positive association with obesity, whereas SCFAs have a negative association with obesity^18^. In addition, the levels of circulating branched-chain amino acids (BCAAs) and glutamate are increased in obese individuals, which is of interest due to the link between glutamate and appetite regulation^19^. The levels of pyrrolidinecarboxylic acid (PA), a glutamate derivative, is increased in the plasma of obese children and is associated with insulin resistance^20^, suggesting that obese individuals consume large amounts of foods that affect protein catabolism and compete with large neutral amino acids for transport into cells in the gut^19^. In addition, research has found that microbial metabolites play a regulatory role in metabolic function through molecular signalling pathway alterations^21^. For instance, microbially produced imidazole propionate impairs insulin signaling by targeting the mTORC1 pathway in type 2 diabetes^21^. However, the causal association between multiple environmental factors and gut microbiota-mediated metabolic function regulation is complex and not yet fully understood.

Low-dose radiation (LDR), which generally refers to radiation at a dose less than 100 millisieverts (mSv), arises from the natural environmental context or from anthropogenic activities, such as nuclear energy production, nuclear waste management, and medical diagnostic and therapeutic procedures. Unlike the well-documented and evident impacts of high dose radiation on biological processes such as DNA damage, carcinogenesis, the effects of LDR are argument. Some studies indicated the promotion of hormesis while some indicated harmful effects^22^. Recently, new evidence suggests LDR’s potential as immune amplifier sensitizing tumors to immune checkpoint blockade responsiveness^23^. Almost 30 million workers are professionally exposed to radiation; of these, interventional fluoroscopists, including cardiologists and radiologists, are among the most highly exposed^24^. A dose of approximately 5 mSv annually is attributed to a projected lifetime cancer risk of 1 in 100 in the following 30 years of work^25^. Considerable uncertainty remains regarding the association of LDR with current best estimates of risks for conditions inducing cancer, diabetes and cardiovascular diseases^26–28^. Among those who are occupationally exposed to radiation in the USA, there isan increased relative risk of diabetes in radiologic technologists^29^. Interestingly, it has been suggested that LDR can alter the gut microbiota and microbial metabolite composition^13,30^. Nonetheless, the specific effects of LDR combined with certain dietary habits remain unclear, and theextent to which the gut microbiota and microbial metabolites derived from LDR or LDR combined with HFD consumption affect metabolic function is largely underexplored.

Given the increasing interest in whether the gut microbiota is linked to metabolic function^15^ in the contexts of radiation exposure, different dietary habits and health conditions, we hypothesized that combining LDR with a diet-related environmental factor, in particular consumption of the common HFD, would promote or ameliorate HFD-induced metabolic impairments. We undertook comprehensive research to test this hypothesis and the underlying mechanisms in mice using multiomics analysis of the gut microbiota, faecal metabolome and plasma metabolome combined with faecal microbiota transplantation (FMT) and antibiotic intervention tests. We showed that LDR worsens the detrimental impact of a HFD on host metabolic function mediated by increased levels of PA and decreased abundance of Parasutterella through the gut microbiota-PA-PYCR1 axis. Our findings suggest that modulation of the gut microbiota, PA and *PYCR1* could be of great value in novel preventative and therapeutic strategies for metabolic diseases.

## Results

### LDR shows potential to aggravate HFD-induced metabolic impairments

**Fig. 1:**
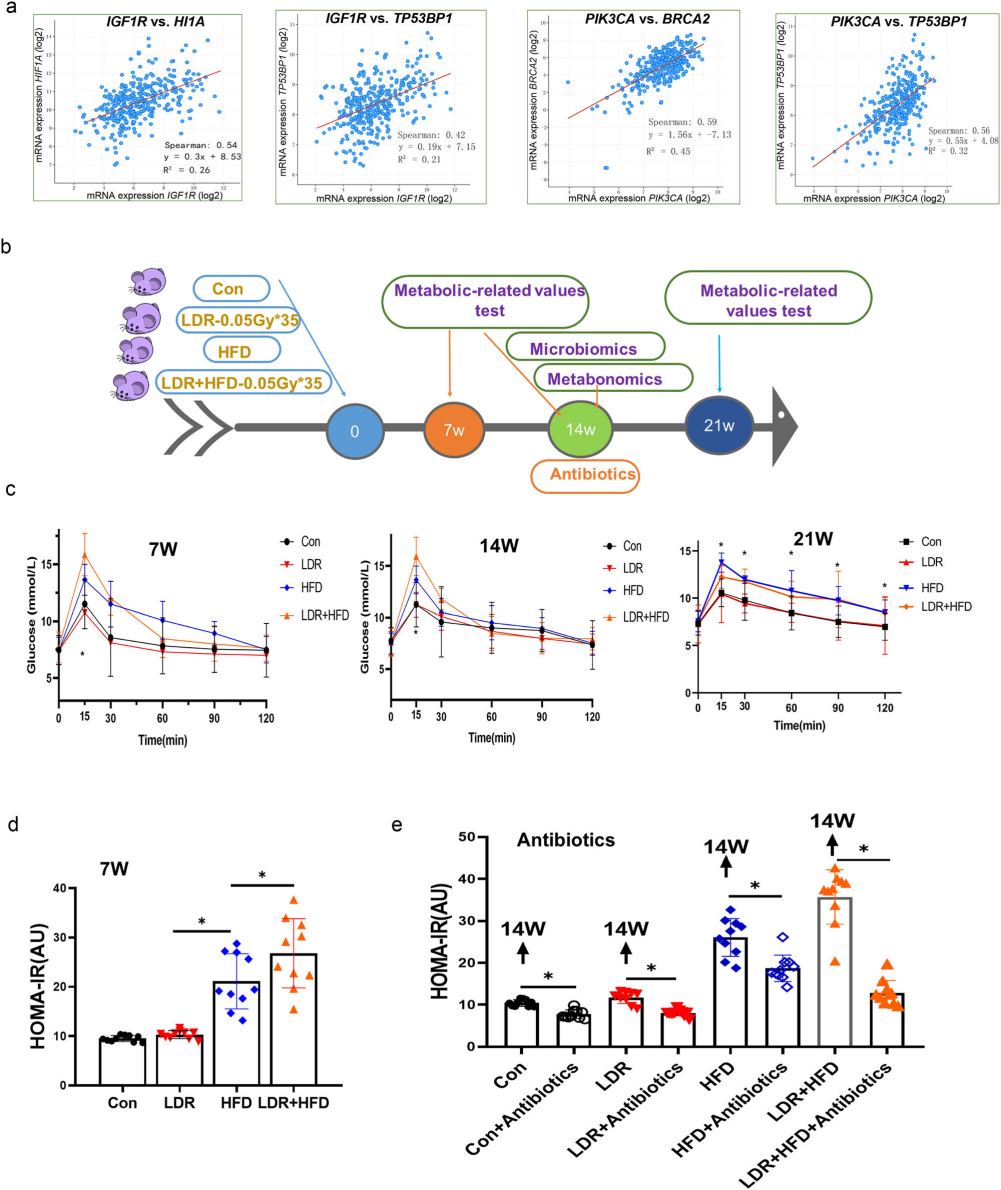
Cancer patients with T2D treated with radiotherapy (RT) may present stronger metabolic impairment effects than those not treated with RT. **a** Correlation analyses of radiotherapy-related genes using the online cBioPortal database (http://cbioportal.org), a resource for exploration, visualization, and analysis of complex cancer genomics and clinical profile data from TCGA. **b** Timeline for the mice subjected to HFD, LDR (0.05 Gy*10) or LDR (0.05 Gy*10) + HFD. **c** Blood glucose levels at the indicated time points among the Con, LDR, HFD and LDR + HFD groups. **d** HOMA-IR values at the indicated time points among the Con, LDR, HFD and LDR + HFD groups. **e** Mice were administered an antibiotic cocktail (50 g/L penicillin G sodium, 50 mg/L metronidazole, 100 U/L streptomycin, and 0.4 g/L neomycin sulfate) in their drinking water for 14 days, after which the mice were subjected to LDR, HFD or LDR + HFD treatment. The mice were killed at 14 weeks, and the HOMA-IR values were detected. The data are the means ± standard deviations (SDs). The Mann-Whitney test or two-tailed unpaired Student’s t-test was used for statistical analyses. *p < 0.05 indicates a significant difference. Error bars indicate SEM (standard error of the mean). **b** was created by Ruixue Huang.

To understand whether there is a potential correlation between ionizing radiation and diabetes, we first compared the mRNA expression levels between cancer radiotherapy resistance-related genes and insulin resistance signalling-related genes from human cancer sample data in The Cancer Genome Atlas (TCGA) using the online cBioPortal database (http://cbioportal.org). *HIF1a*, *Tp53BP1*, and *BRCA2*were correlated with *IGF1R* and *PIK3CA*, with Spearman coefficients of 0.54, 0.42, 0.59, and 0.56, respectively (Fig. 1a, Supplementary Fig. 1a). Based on this investigation, we further investigated whether long-term LDR exposure affects T2D metabolic function. Mice were divided into a control (Con) group, a HFD group, an LDR group exposed to a dose of 0.05 Gy of radiation 10 times, and a LDR and HFD (LDR + HFD) co-exposure group (Fig. 1b, Supplementary Fig. 1b, c). In agreement with a previous report^31^, we observed that the multiple low-dose insults caused the glucose level to be higher in the LDR + HFD group than in the HFD group at the 7^th^ week, 14th week and 21st week (Fig. 1c). The same results were observed for the homeostatic model assessment-insulin resistance (HOMA-IR) value (Fig. 1d). However, no body weight difference was observed between the LDR + HFD group and the HFD group (Supplementary Fig. 1). Antibiotic cocktail treatment among these four groups at the 14^th^ week decreased the HOMA-IR value in the LDR + HFD group compared to the HFD group(Fig. 1e). These results underscore that LDR exposure may be associated with the regulation of metabolic pathways and that this potential association may involve the gut microbiota.

### LDR synergizes with HFD to exacerbate metabolic impairments in mice

**Fig. 2:**
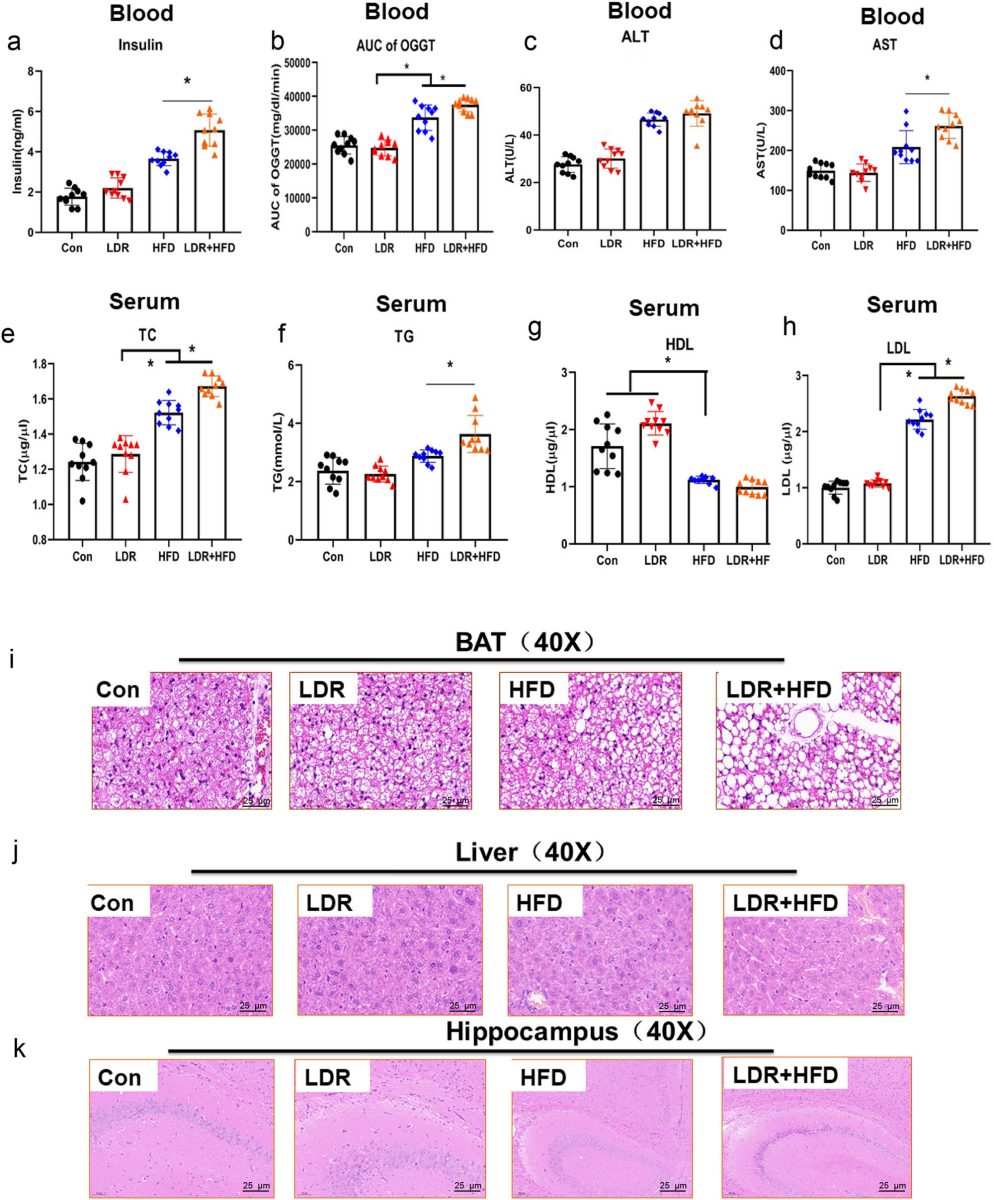
LDR synergizes with HFD to exacerbate metabolic impairments in mice. **a** Blood insulin levels (*n* = 10 per group). **b** Area under the curve (AUC) values from the OGTT of blood samples (2 g/kg mouse). **c** ALT levels of blood samples in mice (*n* = 10 per group). **d** AST levels of blood samples in mice (*n* = 10 per group). **e** TC levels of serum samples in mice (*n* = 10 per group). **f** TG levels of serum samples in mice (*n* = 10 per group). **g** HDL levels of serum samples in mice (*n* = 10 per group). **h** LDL levels of serum samples in mice (*n* = 10 per group). **i** Representative pictures of BAT stained with H&E (40X). **j** Representative pictures of liver tissue stained with H&E (40X). **k** Representative pictures of liver tissue stained with H&E (40X). The data are the means ± the SDs. The Mann-Whitney test or two-tailed unpaired Student’s t-test was used for statistical analyses. *p < 0.05 indicates a significant difference. Error bars indicate SEM (standard error of the mean).

It has been reported that the pancreas, liver, fat issues and brain hippocampus are the major sites influenced by metabolic impairments; thus, we determined the synergistic effects of LDR and HFD on the metabolic parameters of these tissues. First, compared to those in the HFD group, the tissues of the mice in the LDR + HFD group showed more severe damage with irregular edges, atrophy, vacuolization, irregular shapes, irregular arrangement and scattered distribution (Supplementary Fig. 2a). The islet area, α cell mass and β cell mass were higher in the LDR + HFD group than in the Con group, and the islet area and β cell mass were higher in the LDR + HFD group than in the HFD group (Supplementary Fig. 2b–d). We also found that in mice blood samples, the insulin, oral glucose tolerance test (OGGT), liver aspartate transaminase (AST), and serum levels of liver total cholesterol (TC), liver triglyceride (TG), and low-density lipoprotein (LDL) values were altered; specifically, they were higher in LDR + HFD-treated mice than in HFD-fed mice. However, there were no significant differences in alanine transaminase (ALT) and high-density lipoprotein (HDL) between the LDR + HFD and HFD groups(Fig. 2a–h). Furthermore, analysis of brown adiposetissue (BAT), liver and hippocampus histology revealed that BAT was more likely to transform into white fat in the LDR + HFD group and the HFD-fed group than in the Con group (Fig. 2i), whereas hepatic lipid content was higher in the LDR + HFD group than in the HFD-fed group (Fig. 2j). No significant difference in hippocampal histology was observed between the LDR + HFD group and the HFD-fed group (Fig. 2k). Taken together, these results show that LDR potentiates specific aspects of HFD-induced host metabolic syndrome, with notable dysregulation of glucose homeostasis, BAT and liver functions.

### LDR restructures the gut microbiota and microbial metabolites

**Fig. 3:**
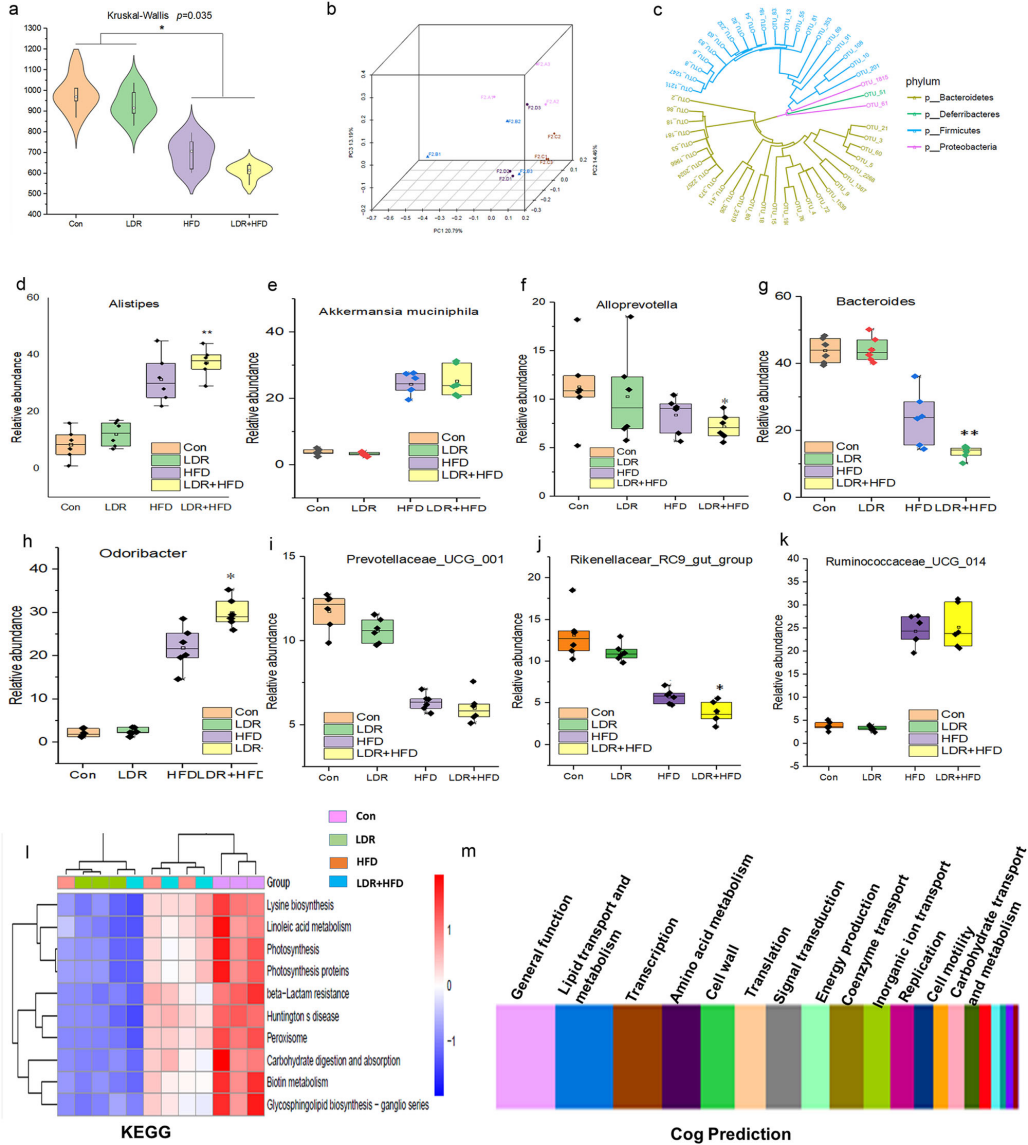
LDR synergizes with HFD to impact the gut microbiome in mice among the Con, LDR, HFD and LDR + HFD groups. **a** Total numbers of observed OTUs in the Con, LDR, HFD and LDR + HFD groups (*n* = 6 mice in each group). **b** PCA based on unweighted UniFrac distances and permutational MANOVA (Adonis) was used to test the differences in gut microbiota composition and diversity among groups. **c** Genus evolutionary tree based on OTUs. **d**–**k** The relative abundance values for different genera were determined by analysis of the composition of the faecal microbiome at 21 weeks. FDR-*p* < 0.05 indicates a significant difference. The Wilcoxon rank-sum test was adopted for comparisons between groups (*p < 0.05 indicates a significant difference). **l** Analysis of the potential KEGG pathways of different representative faecal microbes among the groups at 21 weeks. **m** Clusters of Orthologous Groups of Proteins (COG) prediction of the potential functions of different representative faecal microbes among the groups at 21 weeks. Error bars indicate SEM (standard error of the mean).

The gut intestinal barrier is vital for maintaining gut integrity and permeability to avoid endotoxaemia, such as that caused by lipopolysaccharide (LPS) entry intothe plasma and subsequent low-grade inflammation, which has been considered to play a causal role in the development of noncommunicable diseases^11,32^. For instance, insulin resistance can be triggered by increased inflammation related to a damaged gut barrier^33,34^. Gut microbiota composition was determined from faecal samples of the mice at 21 weeks using bacterial 16 S rRNA gene v3-v4 amplicon sequencing as previously reported^13^. The typical gut microbiota abundance alterations among the four groups are listed in Supplementary Table 1. Specifically, the gut microbiome alpha diversity decreased after co-exposure toLDR and HFD for 21 weeks (Fig. 3a), while the unweighted UniFrac distance of the mice in the LDR + HFD group was different from that of the mice in the other groups, suggesting that LDR may change β diversity(Fig. 3b). To study the phylogenetic relationship, we constructed a phylogenetic tree by combining the base differences of operational taxonomic unit (OTU) sequences at a certain taxonomic level with the species annotation information of each OTU sequence (Fig. 3c). p_Bacteroidetes andp_Deferribacteres had similar evolutionary relationships. LDR treatment in combination with HFD feeding increased Alistipes and Odoribacter abundance, whereas it decreased Bacteroides, Prevotellaceae_UCG_001 abundance (Fig. 3d–k). The Parasutterella abundance was over 10-fold changed in the LDR + HFD group compared to the HFD group (Supplementary Table 1). Moreover, Kyoto Encyclopedia of Genes and Genomes (KEGG) analysis showed that the altered gut microbiota groups were associated with lysine biosynthesis, linoleic acid metabolism and photosynthesis (Fig. 3l) and may participate in lipid transport and metabolism, amino metabolism or signal transduction (Fig. 3m).

**Fig. 4:**
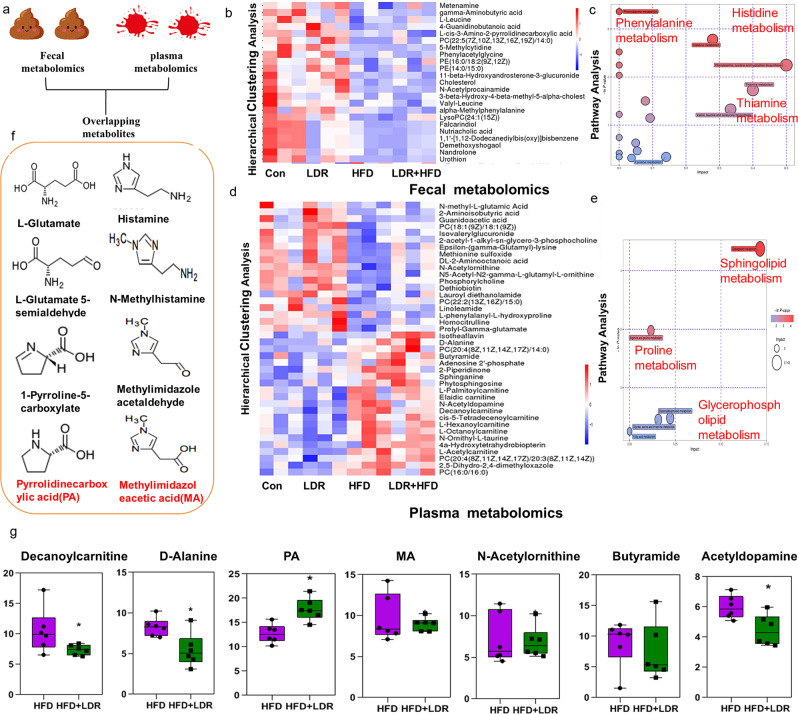
Profiling of faecal metabolomes and plasma metabolomes under conditions of LDR + HFD synergism. **a** Exploration of overlapping metabolites coexisting in both faecal metabolomes and plasma metabolomes. **b** Cluster heatmap showing the metabolite properties of the faecal metabolomes among the Con, LDR, HFD and LDR + HFD groups of mice. **c** The potential metabolic pathways for the mouse faecal metabolomes were analysed among the Con, LDR, HFD and LDR + HFD groups. **d** Cluster heatmap showing the metabolite properties of the plasma metabolomes among the Con, LDR, HFD and LDR + HFD groups of mice. **e** The potential metabolic pathways for the mouse plasma metabolomes were analysed among the Con, LDR, HFD and LDR + HFD groups of mice. **f** The PA and MA pathways were upregulated in the LDR + HFD group compared with the HFD group. **g** Differences in microbial metabolites between HFD and LDR + HFD mice *(n* = 6 mice per group). Microbial metabolites were up- or downregulated in the LDR + HFD group compared with the HFD group. The differences were examined by Wilcoxon rank-sum test (*p < 0.05 indicates a significant difference). The boxplot elements are defined as follows: central line, median; box limits, upper and lower quartiles. Error bars indicate SEM (standard error of the mean). **a** was created by Ruixue Huang and the elements used was permitted by Biorender (https://biorender.com/) because Ruixue Huang is one of the registered user.

LDR + HFD-treated mice demonstrated substantial alterations in the gut microbiota, motivating us to explore whether and how LDR influences gut microbial metabolites and circulating microbial metabolites, whose concentrations are primarily modulated through the gut microbiota. We anticipated that this exploration would offer new insights into the mechanisms underlying the observed phenotypes caused by LDR in combination with HFD feeding with regard to host metabolism. Overall, untargeted metabolomic profiling of faecal and plasma samples revealed marked changes in numerous metabolites after 21 weeks of treatment in the LDR + HFD group compared to the HFD group (Fig. 4a, Supplementary Tables 2 and 3). A cluster heatmap showed that the plasma levels of the metabolites tridecanoic acid, heneicosanoic acid, methylimidazoleacetic acid (MA) and PA were higher in the LDR + HFD group than in the HFD group, whereas the plasma levels of decanoylcarnitine, 4a-hydroxytetrahydrobiopterin, and D-alanine were lower (Fig. 4b). KEGG pathway analysis showed that the altered plasma metabolites in the LDR + HFD group were linked to histidine metabolism, phenylalanine metabolism, and the prolactinsignalling pathway (Fig. 4c, Supplementary Table 4). In addition, a cluster heatmap showed that the levels of the faecal metabolites 5-methylcytidine, urothion, and PA were higher in the LDR + HFD group than in the HFD group, while the levels of I-urobilin, di-4-coumaroylputrescineand fagomine were lower (Fig. 4d). KEGG pathway analysis showed that the altered faecal metabolites in the LDR + HFD group were linked to sphingolipid metabolism, proline metabolism, and glycerophospholipid metabolism (Fig. 4e, Supplementary Table 5). The overlapping metabolites in both faeces and plasma were examined to investigate the link among LDR + HFD treatment, the gut microbiota and the metabolome. Compared to HFD treatment, LDR + HFD treatment resulted in synergistic effects. The levels of the metabolites PA and MA were increased in both faeces and plasma (Fig. 4f, g). The L-glutamate and histamine utilization pathways illustrate that PA and MA synthesis are regulated by a series of enzymes and genes (Fig. 4f). In addition, we assessed the correlations between the gut microbiome and plasma metabolomes to identify the potential microbe-related metabolites. These results indicate that the levels of microbial-related PA in both plasma and faeces may be mediated by the gut intestinal barrier.

Based on these findings, we wanted to explore whether PA-related regulatory enzyme genes may be influenced by LDR. We analyzed certain upstream or downstream metabolites that were linked with PA. The results showed that the levels of L-glutamyl-P, 1-glutamate-5-semialdehyde, and N-acetyl-glutamate-semialdehyde did not differ between the LDR + HFD group and the HFD group (Supplementary Fig. 3a). However, the levels of 1-pyrroline-5-carboxylate and 4-hydroxyproline were higher, while those of 1-Pyrroline 3-hydroxy-5-carboxylate were lower, in the LDR + HFD group than in the HFD group (Supplementary Fig. 3a). Considering that these changed metabolites were regulated by some crucial enzyme-coding genes, including PRODH, P4HA and PRCR1, we then detected the expression of these genes and corresponding inflammation-related cytokines. As shown in Supplementary Fig. 3b, c, compared with HFD treatment, treatment of mice with LDR + HFD increased IFN-γ, TNF-α, NF-KB and PYCR1 levels but decreased PRODH and P4HA levels. We also found that the levels of BAT-related biomarkers, such as Ucp1, DiO2 and Pparα, were decreased in mice treated with LDR + HFD (Supplementary Fig. 3d). These results indicated that the accumulation of PA in plasma may be regulated by the *PRODH* and *PYCR1* genes. We used the GEPIA2 online database (http://gepia2.cancer-pku.cn/#index) to primarily explore the biological functions of *PRODH*, *P4HA, PRODH2* and*PYCR1*. As shown in Supplementary Fig. 4, compared with that in normal tissues, the expression of PRODH and PRODH2 was decreased in various cancer tissues, while that of P4HA and PYCR1 was increased in various cancer tissues. Moreover, pancreatic cancer patients with low PRODH, P4HA and PYCR1 expression had longer survival times than cancer patients with higher expression; in contrast, patients with low PRODH2 expression had shorter survival times than those with high PRODH2 expression (Supplementary Fig. 4). These results indicate that PRODH, P4HA, PRODH2 and PYCR1 may be involved in pancreatic biological functions. Next, PYCR1 was investigated for its potential role in the LDR-PA-PYCR1 axis.

### Gut microbes are required for the aggravating effects of LDR on HFD-induced insulin resistance

To explore the crucial role of the gut microbiota in mediating inherent metabolic impairments, particularly insulin resistance, we investigated whether the observed impairments caused by LDR were influenced by removal of the gut microbiota in HFD-fed mice. Antibiotic cocktail treatment was applied to establish the role of the microbiota in mice treated with LDR + HFD, while LDR + HFD mice treated with probiotics were considered the positive controls (Supplementary Fig. 5a). Antibiotic treatment increased butyrate and propionate concentrations in faeces but reduced plasma LPS, TG and TC levels (Supplementary Fig. 5b–f). Hepatic TG and TC levels decreased in mice treated with antibiotics in the LDR + HFD group (Supplementary Fig. 5g). However, these decreases were reversed in the group treated with probiotics (Supplementary Fig. 5b–g). Haematoxylin (H&E) staining of BAT, the hippocampus and the liver for histological analysis also showed that after antibiotic treatment, the browning of adipose tissues was increased, and liver lipid accumulation was reduced(Supplementary Fig. 5i, j). However, there were no significant pathological changes in hippocampal tissues. We then detected intestinal barrier-related tight junction protein expression by immunofluorescence (IF). As shown in Supplementary Fig. 5k–m, E-cadherin, β-catenin and Occludin expression increased in LDR + HFD-fed mice treated with antibiotics or probiotics. Moreover, transmission electron microscopy (TEM) revealed that antibiotic treatment improved the mitochondrial structure in hippocampal and pancreatic tissues (Supplementary Fig. 6a, b). Considering that DNA damage in the hippocampus was a critical consequence of HFD-induced metabolic impairment, we detected γ-H2AX levels by IF. The results showed that γ-H2AX levels were decreased in both hippocampal and pancreatic tissues. Of note, the insulin level in the pancreas increased after mice were treated with antibiotics or probiotics (Supplementary Fig. 6c–e). We also detected liver inflammation-related cytokine expression and found that IFN-γ, TNF-α, IL-1β, IL-10 and NFKB levels decreased after mice were treated with antibiotics and probiotics (Supplementary Fig. 6c–e). Notably, thermogenic markers, including Ucp1, Cidea, Dio2, pgc1α and Pparα, in brown adipose tissues were dramatically elevated after antibiotic administration in LDR + HFD-treated mice(Supplementary Fig. 6g). In addition, compared with no antibiotic treatment, we found that removal of the gut microbiota in LDR + HFD mice with antibiotics decreased HOMA-IR and fasting glucose levels (Supplementary Fig. 7a, b). Further gut microbiota abundance detection showed that LDR + HFD mice treated with antibiotics had decreased abundance of Lactobacillus, Alistipes, Odoribacter, Lachnospiraceae_NK4A136_group and Parasutterella but increased abundance of Bacteroides (Supplementary Fig. 7c). These results together indicate that removal of the microbiota partly abolishes the aggravating effects of LDR on HFD-induced insulin resistance.

### Effects of HFD and LDR + HFD on FMT-mediated disruption of insulin sensitivity, PA metabolism and PYCR1 expression

**Fig. 5:**
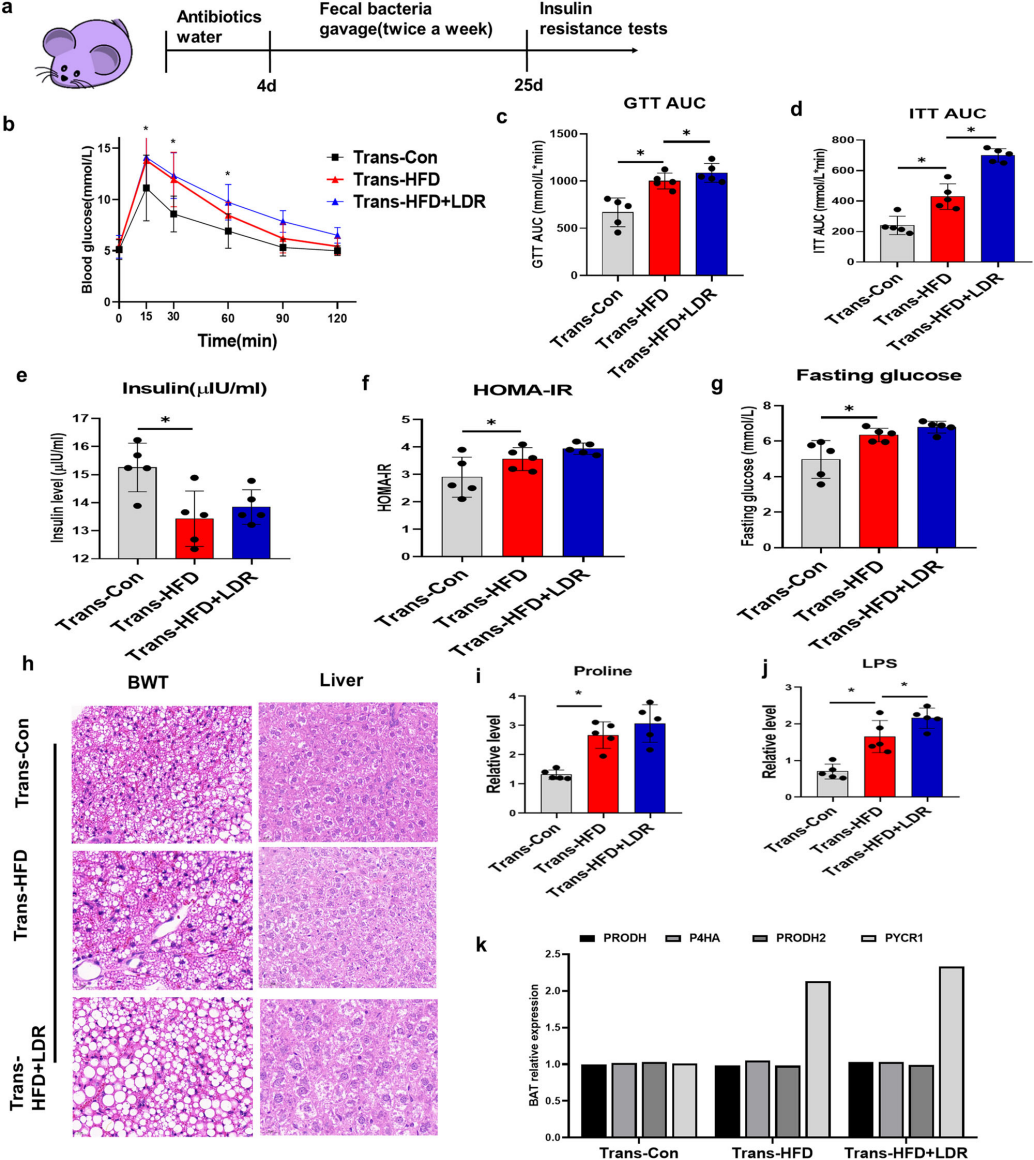
FMT with microbiotas from HFD and LDR + HFD mice disrupts insulin sensitivity, PA metabolism and PYCR1 expression. The mice transplanted with stool suspensions from healthy controls, HFD cases and LDR + HFD cases were defined as Trans-Con, Trans-HFD and Trans-LDR + HFD mice, respectively. After treatment with antibiotics in drinking water for 4 days, the mice were gavaged with stool suspensions twice per week. **a** Timeline for treatment of the mice with faecal bacteria via gavage after antibiotic-mediated depletion of the gut microbiota. **b** Glucose levels (*n* = 5 per group). **c** GTT AUC values (*n* = 5 per group). **d** ITT AUC values (*n* = 5 per group). **e** Insulin levels (*n* = 5 per group). **f** HOMA-IR values (*n* = 5 per group). **g** Fasting glucose values (*n* = 5 per group). **h** H&E staining of representative BAT and liver tissues (40X). **i** Relative levels of PA (*n* = 5 per group). **j** LPS levels (*n* = 5 per group). **k** Relative expression of PRODH, P4HA, PRODH2 and PYCR1 for the Trans-Con, Trans-HFD and Trans-LDR + HFD groups. The data are the means ± SDs. The Mann-Whitney test or two-tailed unpaired Student’s *t*-test was used for statistical analyses. *p < 0.05 indicates a significant difference. Error bars indicate SEM (standard error of the mean). **a** was created by Ruixue Huang and the elements used was permitted by Biorender (https://biorender.com/) because Ruixue Huang is one of the registered users.

To explore the effect of the gut microbiota on insulin resistance in hosts, faeces from healthy controls or mice fed a HFD or subjected to LDR + HFD were transplanted into mice by oral gavage (Fig. 5a). Compared with mice transplanted with faeces from healthy mice, mice transplanted with faeces from HFD- or LDR + HFD-treatedmice displayed insulin resistance, as revealed by glucose levels, glucose tolerance test (GTT) outcomes, insulin tolerance test (ITT) outcomes, insulin levels, HOMA-IR values and fasting glucose levels (Fig. 5b–g). Furthermore, H&E showed that mice transplanted with HFD or LDR + HFD faeces tended to exhibit transformation of BAT into white adipose tissue (WAT), and their liver cells had increased lipid accumulation (Fig. 5h). Further detection of metabolites in plasma revealed that PA and LPS were both increased (Fig. 5I, j). Regarding changes in the relative expression of PA-regulating genes, only PYCR1 exhibited elevated expression in mice transplanted with HFD or LDR + HFD faeces; other genes, including PRODH, P4HA, and PRODH2, did not exhibit changes (Fig. 5k).

### PA administration aggravates HFD-induced gut barrier damage and insulin resistance

**Fig. 6:**
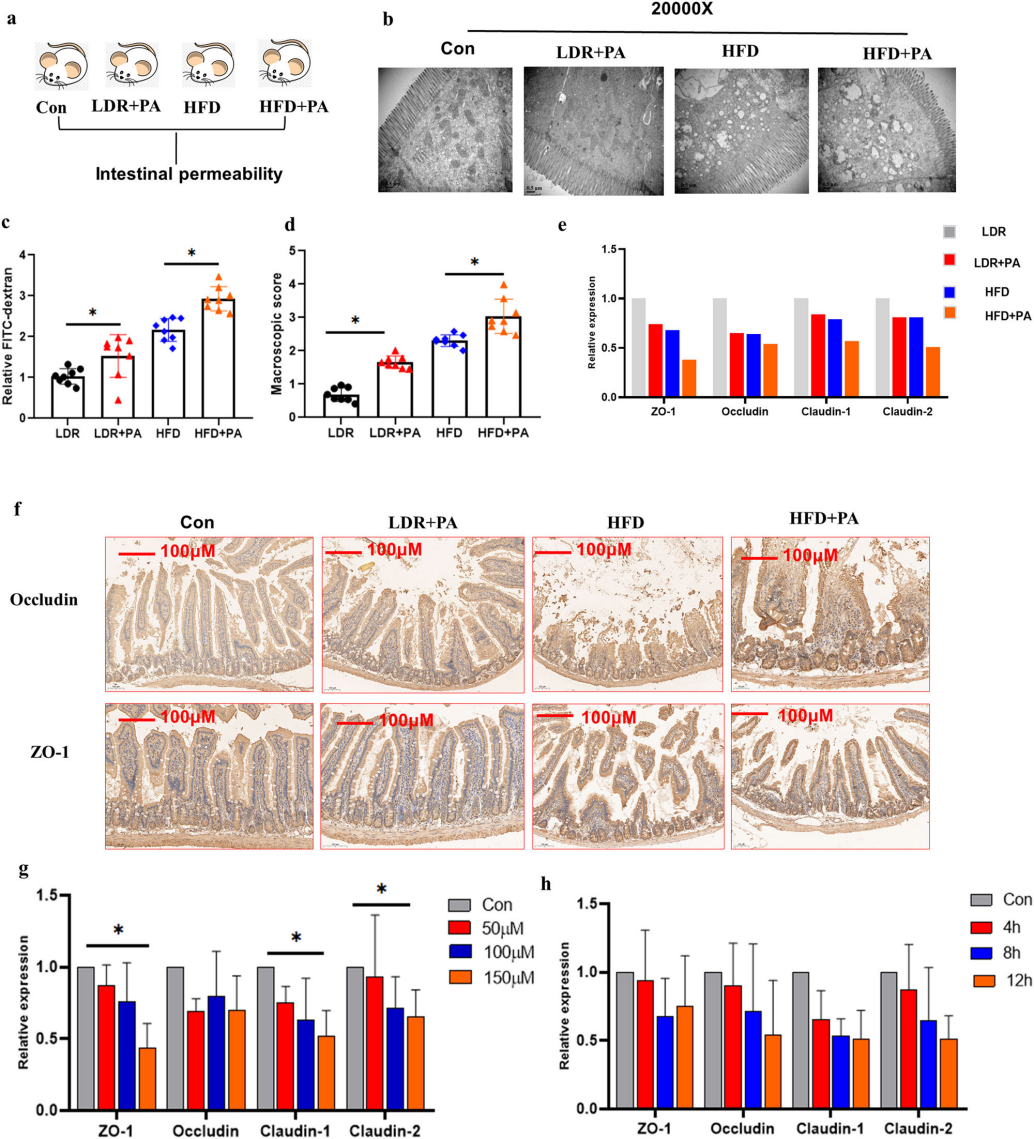
PA promotes HFD-induced intestinal impairments. Mice were divided into four groups: the Con, LDR (0.05 Gy*10) +PA, HFD, and HFD + PA groups. **a** Trial design for exploration of the effects of PA on mice. **b** Representative electron microscopy images of intestinal structure among the four groups. 20000X. **c** Relative FITC-dextran levels. **d** Macroscopic scores. **e** mRNA expression of the intestinal tight junction genesZO-1, Occludin, Claudin-1 and Claudin-2 among the four groups. **f** Immunohistochemical detection of Occludin and ZO-1 in intestinal tissues. Scale bar: 100 µm. **g** The HCT116 cell line was used to detect theeffects of PA on intestinal tight junction gene mRNA expression at the indicated PA concentrations. **h** The HCT116 cell line was used to detect the effects of PA on intestinal tight junction gene mRNA expression at the indicated PA treatment timepoints. The data are the means ± SDs. The Mann-Whitney test or two-tailed unpaired Student’s *t*-test was used for statistical analyses. *p < 0.05 indicates a significant difference. Error bars indicate SEM (standard error of the mean). **a** was created by Ruixue Huang and the elements used was permitted by Biorender (https://biorender.com/) because Ruixue Huang is one of the registered user.

**Fig. 7:**
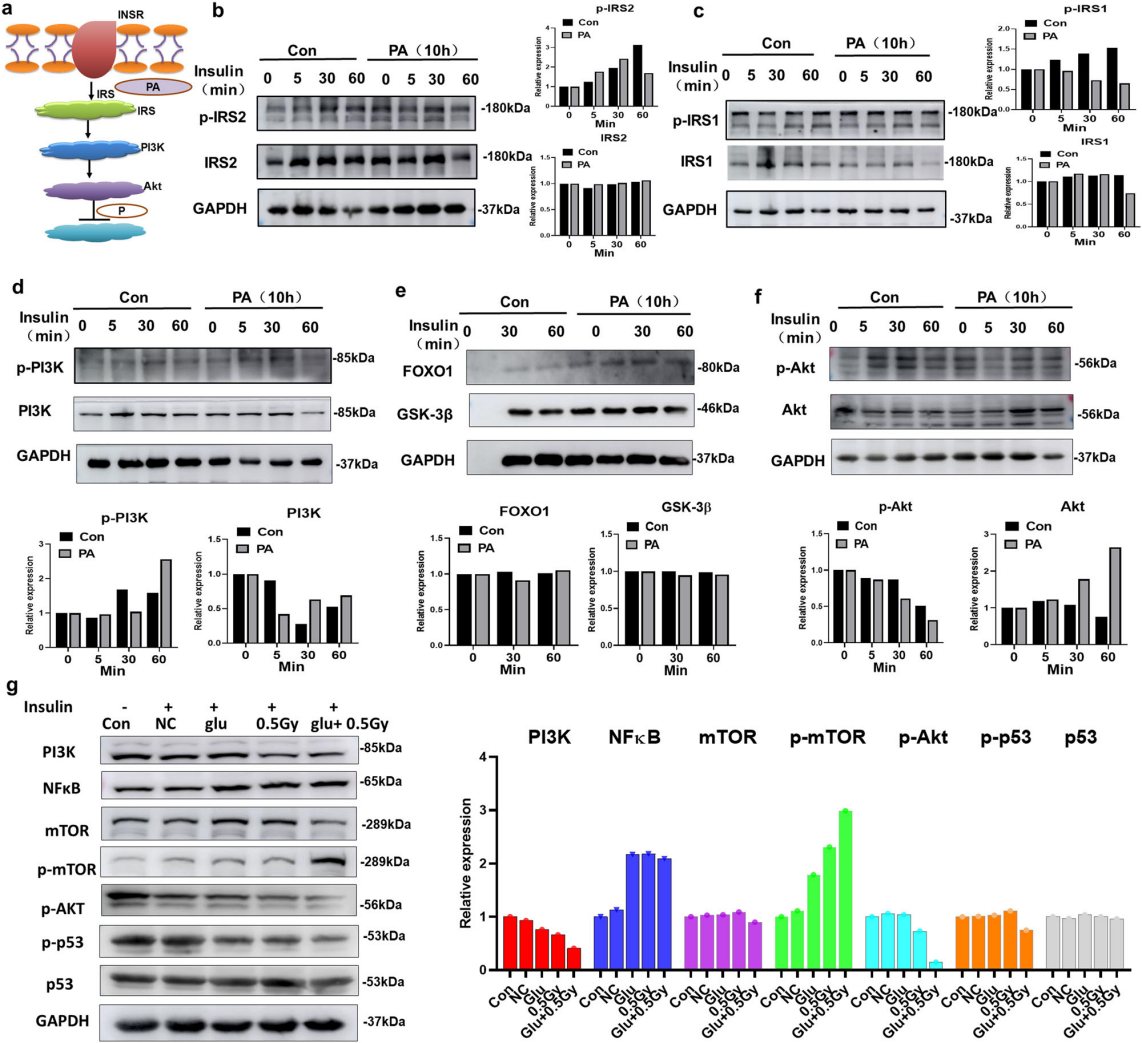
PA promotes HFD-induced insulin resistance in liver cells. **a** Hypothesis of the potential underlying mechanism regarding the effects of PA on the IRS/Akt signalling pathway. **b** Western blot analysis (and quantification) of the effects of PA (100 μM for 10 h) on insulin-stimulated p-IRS-2 and IRS2 expression in HepG2 cells. **c** Western blot analysis (and quantification) of the effects of PA (100 μM for 10 h) on insulin-stimulated p-IRS-1 and IRS1 expression in HepG2 cells. **d** Western blot analysis (and quantification) of the effects of PA (100 μM for 10 h) on insulin-stimulated p-PI3K and PI3K expression in HepG2 cells. **e** Western blot analysis (and quantification) of the effects of PA (100 μM for 10 h) on insulin-stimulated FOXO1 and GSK-3β expression in HepG2 cells. **f** Western blot analysis (and quantification) of the effects of PA (100 μM for 10 h) on insulin-stimulated p-Akt and Akt expression in HepG2 cells. **g** Western blot analysis (and quantification) of the effects of co-exposure to glucose and LDR (0.5 Gy) on insulin-stimulated PI3K, NFκB, mTOR, p-mTOR, p-Akt, p-p53, and p53 expression in HepG2 cells with or without glucose and LDR (0.5 Gy) treatment. GAPDH was used as the internal reference. The data are the means ± SDs. The Mann-Whitney test or two-tailed unpaired Student’s *t*-test was used for statistical analyses. *p < 0.05 indicates a significant difference. Error bars indicate SEM (standard error of the mean). **a** was created by Ruixue Huang and the elements used was permitted by Biorender (https://biorender.com/) because Ruixue Huang is one of the registered user.

An additional animal study was performed to investigate the roles of LDR-regulated microbial metabolites, particularly PA (whose levels were increased in the antibiotic-administered mouse group), in regulating HFD-induced gut intestinal barrier damage as well as insulin resistance-related signaling pathways (Fig. 6a). Compared with the Con mice, HFD mice administered PA showed markedly reduced mitochondrial numbers and mitochondrial swelling, which perturbed proper mitochondrial function in the LDR + PA and HFD + PA groups (Fig. 6b). Notably, FITC-dextran and microscopic scores were also increased in HFD mice administered PA compared to HFD mice (Fig. 6c). The gutintestinal barrier-related tight junction proteins ZO-1, Occludin, Claudin-1 and Claudin-2 were reduced in HFD mice administered PA compared to the HFD mice (Fig. 6d). Immunofluorescence detection showed that the expression of Occludin and ZO-1 was lower in HFD mice administered PA than in the HFD mice (Fig. 6f). We then detected whether a dose-dependent and time-dependent relationship existed between the relative expression of gut intestinal barrier-related tight junction proteins and PA using the HCT116 cell line. As shown in Fig. 6g, h, as the PA dosage and time treatment increased, the relative expression of these genes also gradually decreased. These data showed that PA intervention had a synergistic regulatory effect on HFD-induced gut intestinal barrier damage. Since previous reports have identified that gut intestinal barrier damage may cause increased plasma LPS and inflammation-related metabolic impairment^35^, we speculated that PA may inhibit insulin signalling. Then, we detected insulin resistance signalling using the HepG2 and 3T3-L1 cell lines, focusing on the IRS1/Akt/mTOR signalling pathway (Fig. 7a). In agreement with our hypothesis, we found that (i) PA decreased p-IRS2, IRS2, p-IRS1, p-PI3K, PI3K, and p-Akt expression but increased Akt expression and had no influence on FOXO1 and GSK-3β (Fig. 7b–f); (ii) compared to the cells that received 0.5 Gy of radiation, those that received glucose+0.5 Gy exhibited reduced PI3K, NFkB, mTOR and p-Akt expression but no alterations in the expression of p53, which can activate PRODH (Fig. 7g)^36^. These data indicate that PA can act as an IRS-1/Akt signalling inhibitor under physiological conditions in vivo through inhibition of PYCR1 activity.

### The PA-IRS-1/Akt pathway regulates PYCR1-aggravated insulin resistance in LDR + HFD mice

**Fig. 8:**
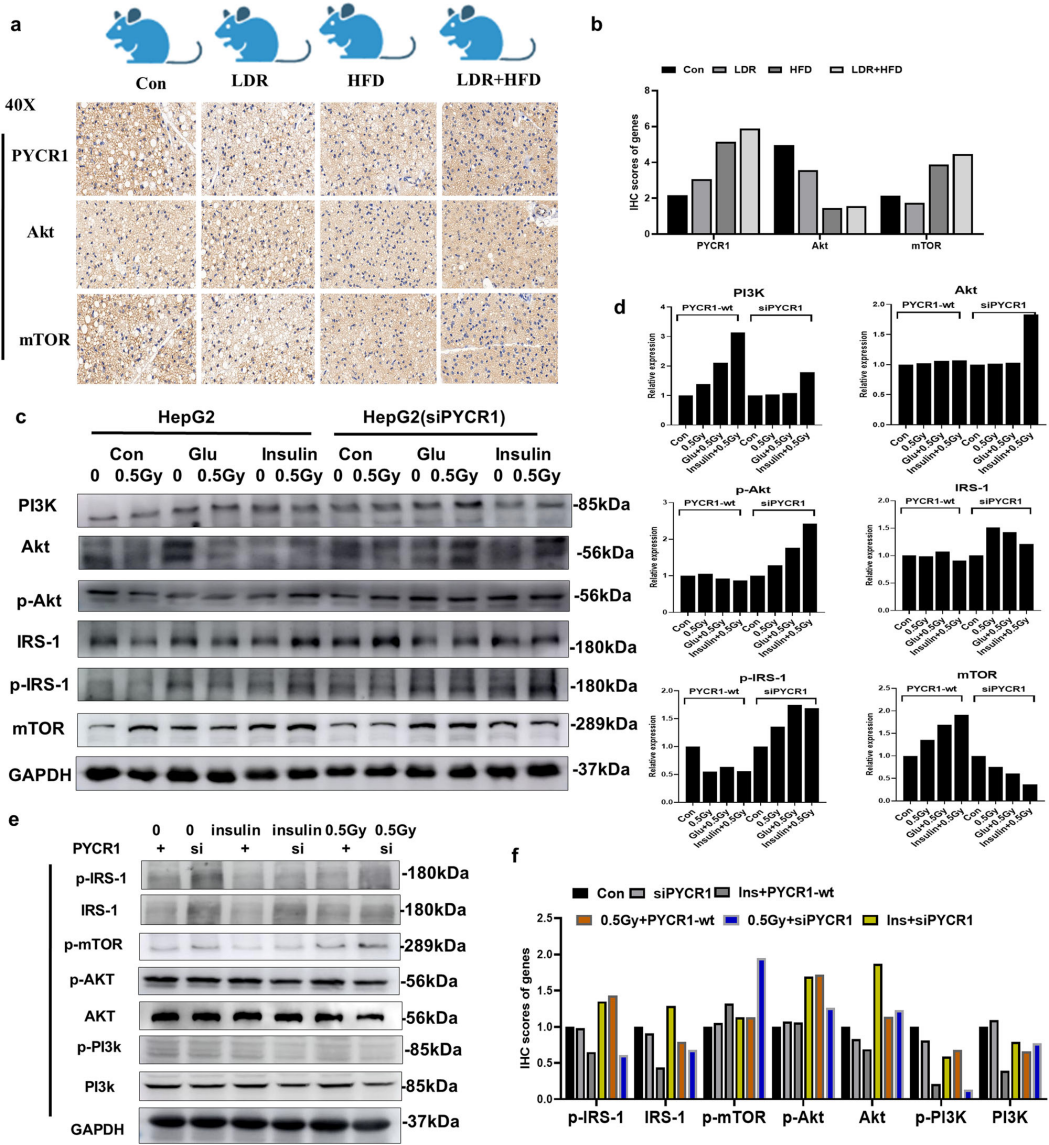
Suppression of the PA-mTOR pathway-regulated molecule PYCR1 improves insulin resistance induced by co-exposure to glucose and radiation in liver cells and adipose cells. **a** Mice were divided into four groups: the Con, LDR (0.05 Gy*10), HFD, and LDR + HFD groups.Immunohistochemical detection of PYCR1, Akt and mTOR in liver tissues. 40X. **b** Quantification of relative PYCR1, Akt and mTOR expression. **c**, **d** HepG2 cells were treated with or without siPYCR1. Western blotting (**c**) and quantification of PI3K, Akt, p-Akt, IRS-1, p-IRS-1, and mTOR protein expression (**d**) were performed for HepG2 cells co-exposed to glucose and LDR (0.5 Gy). Western blot analysis (**e**) and quantification (**f**) of the effects of co-exposure to glucose and LDR (0.5 Gy) on insulin-stimulated p-IRS-1, IRS-1, p-mTOR, p-Akt, Akt, p-PI3K and PI3K expression in 3T3-L1 cells. GAPDH was used as the internal reference. The data are the means ± SDs. The Mann-Whitney test or two-tailed unpaired Student’s t-test was used for statistical analyses. *p < 0.05 indicates a significant difference. Error bars indicate SEM (standard error of the mean). **a** was created by Ruixue Huang and the elements used was permitted by Biorender (https://biorender.com/) because Ruixue Huang is one of the registered user.

The possible mechanisms by which PYCR1 regulates insulin resistance in LDR + HFD mice were investigated. First, we found that PYCR1 and Akt expression was higher, while mTOR expression was lower, in mice treated with LDR + HFD than in HFD mice (Fig. 8a, b). We then knocked down the expression of PYCR1 in HepG2 cells. Further study indicated that PYCR1 deficiency increased PI3K, IRS-1 and p-IRS-1 expression but decreased mTOR expression in HepG2 cells without PYCR1 expression; in addition, PYCR1 deficiency improved insulin resistance mediated by combined glucose/0.5 Gy treatment due to increases in p-Akt, IRS-1 and p-IRS-1 but a decrease in mTOR compared with the levels in HepG2 cells treated with glucose and 0.5 Gy without PYCR1 deficiency (Fig. 8c, d). Moreover, in adipose cells, PYCR1 deficiency combined with 0.5 Gy treatment increased Akt and PI3K expression but increased p-mTOR expression compared with the levels in 3T3-L1 cells treated with 0.5 Gy without PYCR1 deficiency (Fig. 8e, f). These data indicated that PYCR1-induced insulin resistance was associated with PA-IRS-1/Akt signalling inhibition. Although the expression of some insulin resistance-related proteins was not altered in PA-treated or PYCR1-deficient liver or adipose cells, our data partly elucidate the causation of, and the roles of the gut-PA-PYCR1 axis in, the aggravating effect of LDR on HFD-induced insulin resistance.

## Discussion

LDR has dual effects (beneficial and non-beneficial) on diabetes or diabetes-induced brain damage^37–40^, and its complications may occur via restructuring of the gut microbiota and microbial-derived metabolites^30,41^. However, the impact of LDR on HFD-associated obesity and metabolic dysfunction and whether and how its expansion could play roles remain poorly understood, highlighting an urgent need to investigate the underlying mechanisms. Here, we carried out a hypothesis-driven approach using a combination of analyses of the host microbiome and metabolome to dissect how LDR is able to aggravate the host metabolic response to HFD. Our results showed that in addition to intestinal proinflammatory effects, LDR promotes intestinal barrier defects, proline dysmetabolism, alterations in the gut microbiome and faecal and plasma metabolome functional profiles, leading to the worsening of HFD-induced obesity and insulin resistance effects. Mechanistically, increased PA levels in plasma can induce liver PYCR1 expression to further inhibit the IRS-1/Akt/mTOR signalling pathway and thus exacerbate HFD-induced obesity and metabolic dysfunction.

Previous studies on the effects of obesity and metabolic damage have produced conflicting results. One epidemiological study showed that chronic LDR exposure increased the occurrence of obesity and hypercholesterolemia among those involved in clean-up after the Chernobyl Nuclear Power Plant accident^42^. In contrast, exposure of mice with HFD-induced diabetic kidney disease to LDR from 25 mGy to 75 mGy can improve lipid profiles, insulin sensitivity and attenuate inflammation and oxidation after 4 weeks, but the beneficial effects are weakened after 8 weeks of LDR exposure^43^. Furthermore, exposure of medaka fish to LDR at 2.25 mGy to 204.3 mGy per day for 190 days has been found to alter glycoprotein glycosylation^44^, but a cell study has revealed that LDR induces hormesis and an adaptive response in normal cells but not in cancer cells, indicating that LDR has distinct biological effects on normal and cancer cells due to different molecular mechanisms^45^. Moreover, exposure of adipose-derived stem cells to LDR(1.4 Gy/min) slows the proliferation rate and causes cell cycle arrest^46^, and exposure to 10 cGy to 50 cGyLDR causes DNA damage in human adipose mesenchymal stem cell nuclei^47^ or activation of the NLRP3 inflammasome inhuman lung cells^48^. In adipose cells of breast cancer tissues, LDR can activate inflammatory cytokines, the levels of which are elevated following LDR exposure^49^. Although Vibe N et al. found that exposure of L6 cells to 1–2 Gy decreased insulin signalling in radiated preadipocytes^31^, there have been limited cell studies on the effects of LDR on insulin resistance in adipose cells and liver cells. Our study results are consistent with those of studies on long-term HFD consumption indicating that chronic exposure to LDR aggravates symptoms of HFD-induced metabolic dysfunction, such as insulin resistance. Possible explanations for differences in results include the dosage selection and mouse and cell line selection in our study. Indeed, in this study, we mimicked occupational LDR exposure in the real world in mice; the mice were treated chronically with multiple LDR exposures every three days for 35 times. Therefore, our results suggest that in the real world, workers with occupational LDR exposure have increased risks of insulin resistance or metabolic dysfunction if they also consume a HFD. Further study is needed to determine the effects of LDR on glucose metabolism and insulin resistance in other diabetes mouse models or other metabolic dysfunction mouse models.

A critical clue regarding the strong links among the alterations in circulating microbial metabolites and the LDR-induced changes in the microbiome profile and HFD-associated metabolic dysfunction was revealed by integrated faecal microbiomics, faecal metabolomics and plasma metabolomics. The effect of LDR on the gut microbiota has been studied previously^13,30,50^. We previously reported that a dose of less than 0.5 Gy of radiation increases Clostridium, Helicobacter and Oscilibacter abundance in mice^13^. David C et al reported that mice exposed to 0.1 and 0.25 Gy of radiation had increased*Verrucomicrobia*^30^.

Radiologists are the typical represented population for exposure of LDR. A study showed that 7.1% of the enrolled radiologists had metabolic syndrome and 58.6% had at least one pathological components including obesity, hypertension, elevated cholesterol level and hyperglycemia^51^. Another study showed that the prevalence of diabetes and obesity among healthy decontamination workers employed after the Fukushima nuclear disaster were 11.3% and 49.0%, respectively^52^. Considering HFD is a popular dietary habit currently, herein, we observed that chronic LDR exposure promoted HFD-induced gut barrier damage and increased plasma LPS concentrations and inflammatory cytokine levels, which can partly illustrate how LDR exacerbates inflammatory responses. LDR also changed microbiome diversity in HFD-fed mice, and the altered diversity was accompanied by an increase in the abundance of Parasutterella, which is inconsistent with the findings of a previous study. We think this inconsistency is partly because the LDR-induced gut microbiota restructuring might have varied due to animal stain diversity, feeding conditions, baseline gut microbiota conditions and the intervention duration. To further explain how LDR aggravates metabolic dysfunction, we investigated the overlapping metabolites in both the gut and plasma, seeking to further identify the role of the gut barrier in regulating crucial metabolites such as SCFAs, LPS and amino acids. In this study, we observed that LDR increased both faecal and plasma levels of microbial metabolites, particularly PA, a glutamate derivative, suggesting that proline metabolism plays an important role in the gut microbiota-metabolite-liver axis in relation to insulin resistance in HFD-treated mice. In line with our study, a previous study revealed that mice exposed to 0.5 Gy and 2 Gy of LDR had changed amino acid metabolism, including tryptophan and proline metabolism^53^. Another study also found a positive dose-response relationship between radiation and proline levels^54^. Proline metabolism dysregulation has been found to be associated with obesity^20^, colon cancer^55^,and dementia^56^. A study has also reported that the level of PA is increased in plasma in the context of obesity but that it is decreased after dietary intervention using black soybean peptides for 12 weeks^57^. Furthermore, in a recent study, PA was associated with an increased risk of incident T2DM^58^. In addition, proline metabolism homeostasis is essential for glucose homeostasis^59^ and neurological dysfunction^60^, which might partly explain why insulin resistance-related proteins and inflammation-related cytokines were altered in LDR + HFD mice compared to HFD mice. These data also indicate that the impact of LDR on proline metabolism may be one mechanism by which LDR affects the gut microbiota to potentiate HFD-induced metabolic impairment, particularly gut barrier dysfunction, inflammation and insulin resistance.

The IRS-1/Akt/mTOR signalling pathway plays an important role in sustaining host glucose homeostasis^61^. We found that LDR aggravated HFD-induced insulin resistance, consistent with inhibition of PI3k/Akt/mTOR signalling although activation of the expression of PYCR1, a key protein that promotes PA synthesis and regulates proline metabolism^62^. PYCR1 has been reported to be associated with the insulin signalling pathway and to inhibit cell proliferation in liver cancer cells^63^. PYCR1, a key enzyme in mitochondrial proline biosynthesis, is upregulated in liver cancer in both humans and animal models^64^. Herein, we observed that LDR strongly inhibited HFD-induced insulin resistance through PYCR1 activation to regulate insulin signalling, which is consistent with a previous report that inhibition of Akt/mTOR can induce insulin resistance^65^. Furthermore, the non-beneficial effects of LDR-mediated PYCR1 activation on insulin resistance were reversed after knockdown of PYCR1 expression in liver and adipose cells, indicating the key role of PYCR1 and its derived downstream insulin signalling pathway in mediating HFD-induced metabolic dysfunction. Mitochondrial number and structure in the intestine and liver were improved by antibiotic and probiotic treatment, which further confirmed that the gut microbiota and gut barrier were highly correlated with the expression of the liver mitochondrial gene PYCR1. These results indicate that (i) the effects of antibiotic and probiotic treatment in the context of combined LDR + HFD-related insulin resistance need to be further investigated and that (ii) the role of PYCR1 in mediating Akt/mTOR signalling in LDR + HFD-induced metabolic dysfunction needs to be confirmed.

**Fig. 9:**
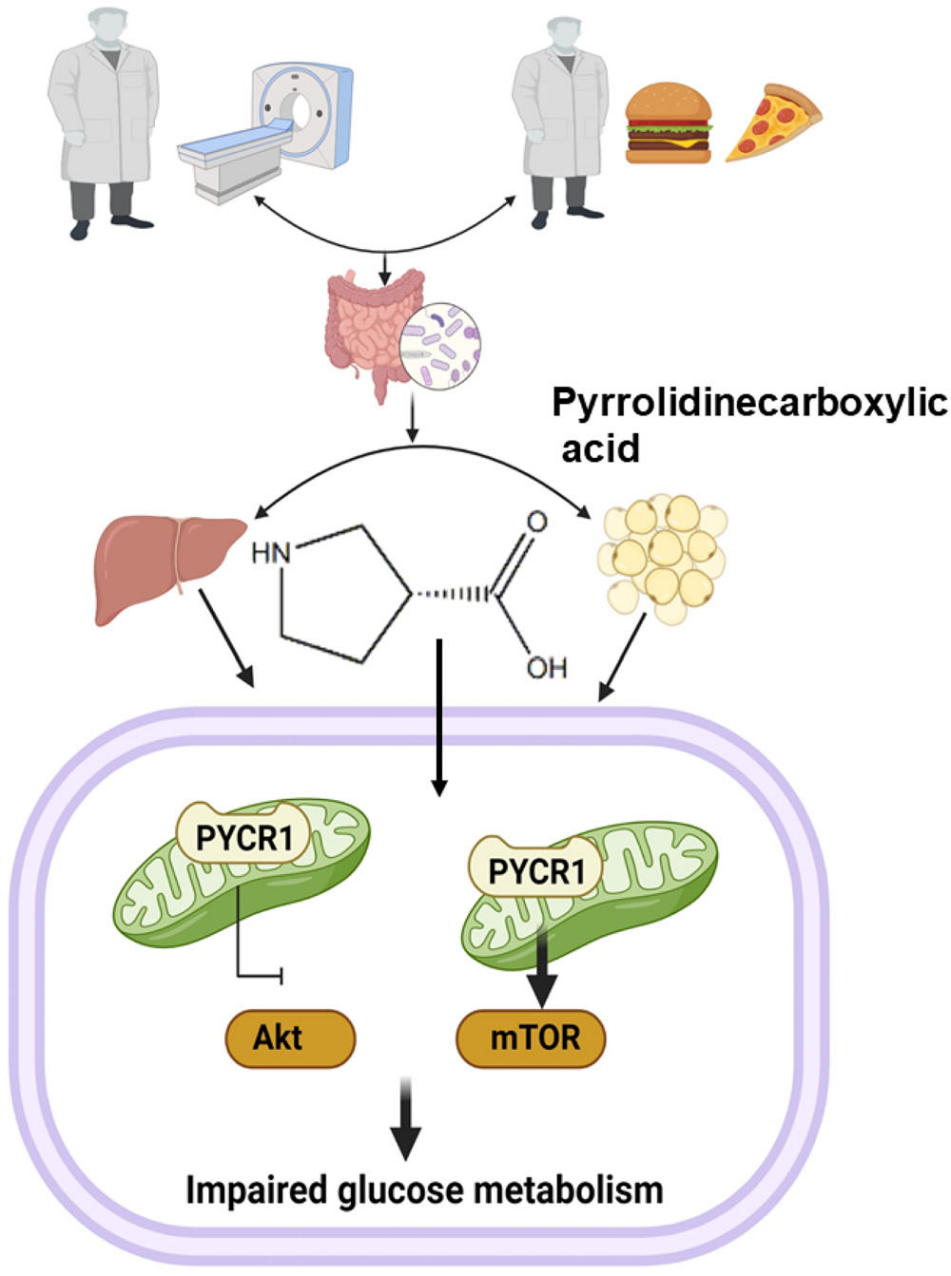
Flowchat of potential mechanism of this study. Figure was created by Ruixue Huang and the elements used was permitted by Biorender (https://biorender.com/) because Ruixue Huang is one of the registered user.

Overall, we have demonstrated a critical role for LDR in promoting HFD-induced intestinal barrier damage, metabolic dysfunction and insulin resistance. This work further emphasizes the importance of the plasma metabolite PA and its interaction with innate insulin resistance within the context of the PYCR1 inhibition-mediated Akt/mTOR signalling involved in the manifestation of LDR + HFD-associated metabolic impairment (Fig. 9). Future investigation is needed on several topics. First, increased attention should be given to studying the risks of LDR. Although attention has previously been given to LDR-related cancer risks, increased focus is needed on the risks to multiple other organs and systems, such as the endocrine system and cardiovascular system. As our mechanism flowchart shows, LDR exposure can be high in certain contexts; alternatively, damage can accumulate (low means greater damage) to finally induce health effects. Second, because PA-mediated Akt/mTOR inhibition might be the link between the gut microbiota and LDR + HFD-induced metabolic impairment, it is necessary to further study the host metabolic impact of blocking or enhancing PA production in bacteria to test this possibility. Of note, PA-based targets bind to potential regulatory gene targets, suggesting that other signalling pathways can also be triggered by PA; therefore, further identification of signalling pathways induced by PA may reveal more intervention targets that can be used to treat HFD-related diseases featuring altered microbial metabolism.

## Methods

### Radiation, chemicals and cell lines

The radiation source was provided by the Institute of Radiation Medicine, Academy of Military Medical Sciences(Beijing, China). The dosage was 0.5 Gy of 60Co γ radiation administered to mice 10 times at room temperature, and 0.5 Gy or 4 Gy was administered to cells at room temperature. PA was purchased from Sigma (CAS No. 147-85-3). The antibodies used for Western blot analysis targeted the following proteins: GAPDH(CST, 5174), E-cadherin (CST, 14472), PI3K (Abcam, ab73262), Akt (Proteintech, 10176-2-AP), p-Akt (Abcam, ab81283), IRS-1 (CST, 2382), p-IRS-1 (CST, 2381), mTOR (Bioworld, bs1844), p-mTOR (CST, 5536 S), PYCR1 (Abcam, ab279385), p53 (CST, 2527 S), p-PI3K(CST, 4228 T), Occludin (Abcam, ab216327), ZO-1 (Abcam, ab221547), Claudin-1 (Abcam, ab211737), CLDN (Abcam, ab207300), N-cadherin (CST, 31593), IFN-γ (Abcam, ab224197), MLCK(Abcam, ab76092), IL-17A (Abcam, ab79056), p-ERK(Abcam, ab279084), GSK3β (Abcam, ab32391), INSR (CST, 23413), BAD (Abcam, ab32445), and TC10 (Abcam, ab32079). An antibiotic cocktail including ampicillin sodium salt (CA2031-5G), polymyxin B sulfate salt (CP8711-100MG) and streptomycin sulfate(CS 10481-5 G) was purchased from Coolaber Science & Technology. The probiotics included *Lactobacillus rhamnosus*(No. CICC 20259) and *Lactobacillus reuteri* (No. CICC 6118), which were purchased from the China Center of Industrial Culture Collection (http://www.china-cicc.org/).PA was purchased from Solarbio (p0011). Insulin was purchased from CoolaberScience & Technology (CI6561), China.

Three types of cells were used in the study: HepG2, HCT116 and 3T3-L1 cells. The HepG2 cells were purchased from the Cell Bank of Type Culture Collection of the Chinese Academy of Sciences (Shanghai, China). The HCT116 and 3T3-L1 cells were purchased from the American Type Culture Collection (ATCC). The HepG2 cells were cultured in Dulbecco’s modified Eagle’s medium(DMEM; HyClone, Utah, USA) with 10% foetal bovine serum (FBS) and 1% antibiotic in a 5% CO_2_ cell incubator at 37 °C as described in our previous reports^66^. The HCT116 cells were cultured in RPMI 1640medium supplemented with 10% FBS and 1% penicillin/streptomycin at 37 °C in a humidified atmosphere with 5% CO_2_. The 3T3-L1 cells were cultured in DMEM containing 10% FBS at 37 °C and 5% CO_2_. For adipogenesis, 3T3-L1 cells were incubated with differentiation cocktail (DMI) medium(Sigma-Aldrich, USA), which included 0.5 mM 3-isobutyl-1-methylxanthine (IBMX) (Sigma, USA), 1 μM dexamethasone (Sigma, USA) and 10 μg/mL insulin (Sigma, USA), as previously reported^67^.

### Mice, antibiotic/probiotic treatment and FMT

Male C57BL/6 mice were purchased at 8 weeks of age from the Fengtai Animal Center, Beijing, China. All of the mice were housed at the Animal Laboratory Division, Beijing Key Laboratory for Radiobiology, Beijing Institute of Radiation Medicine, Academy of Military Medical Sciences (AMMS), Beijing, China. All animal procedures and testing were conducted according to the National Legislation and local guidelines of the Laboratory Animals Center at the AMMS. The study and research protocols were approved by the Institutional Animal Review Board of Central South University (2020sydw0110). In addition, all animals in the study were treated humanely with regard to the alleviation of suffering. All animal procedure experiments were approved by the Committee on Animal Use and Care of Central South University, China. All the mice were maintained in a specific-pathogen-free (SPF) environment with controlled conditions of a 12 h light/dark cycle at 20–22 °C and 45 ± 5% humidity. After 1 week of acclimation, the mice were used for the study with the group design detailed below.

To investigate the effects of LDR on HFD-induced metabolic impairments, mice were divided into four groups: the Con group, the LDR group, the HFD group and the LDR + HFD (co-exposure) group. The mice in the Con group were fed ad libitum with a conventional diet. The mice in the LDR group were exposed to 0.05 Gy of radiation every three days 35 times; this dosage was selected according to a previous report indicating that a dose between 0.05 mGy and 20 mGy is comparable to the dose limit for radiation workers^68^ as well as our previous study^13^. The mice in the HFD group were fed a HFD (38% fat, dominantly milk fat), whereas those in the LDR + HFD group were exposed to 0.05 Gy of radiation 10 times and fed a HFD. Body weight, food intake and water consumption were recorded. The mice were killed at 21 weeks to collect serum and tissues for further detection of various parameters.

For investigation of the gut microbiota-dependent effects, mice were divided into four groups: the Con group, the LDR + HFD group (in which mice were co-exposed to 0.05 Gy of radiation every three days 35 times and fed a HFD), the LDR + HFD + antibiotics group (in which LDR + HFD mice were treated with an antibiotic cocktail that included 50 g/L ampicillin sodium, 100 U/L streptomycin, and 50 mg/L polymyxin B sulfate, as previously described^69^) and the LDR + HFD + probiotics group (in which mice were treated with probiotics with dosages chosen based on our previous report^70^). Briefly, probiotics, including *Lactobacillus rhamnosus*(No. CICC 20259) and *Lactobacillus reuteri* (No. CICC 6118), were purchased from the China Center of Industrial Culture Collection (http://www.china-cicc.org/). The probiotics were administered to the mice orally after being dissolved in PBS solution containing 50 × 10^9^ CFU of probiotics. The mice were administered antibiotics and probiotics prior to LDR + HFD co-exposure for 2 weeks, and then the experiment was performed for 14 weeks. The mice were killed at 21 weeks to collect serum and tissues for further detection of various parameters.

To investigate the effects of PA, mice were divided into four groups: the Con group, the LDR + PA group (in which mice were co-exposed to 0.05 Gy of radiation every three days 35 times and fed PA purchased from Sigma (CAS No. 147-85-3)), the HFD group, the HFD + PA group (in which mice were fed a HFD and 50 mg/kg PA). The mice were treated for 12 weeks and then killed to collected tissues for detection of various parameters.

For the FMT study, the microbiota donors were mice treated with a HFD or LDR + HFD for 10 weeks. Faeces of the donors were collected and dispersed in sterile Ringer working buffer, and the supernatant was mixed with skimmed milk for transplantation. Four-week-old germ-free male C57BL/6 J mice were randomly divided into three groups (with 10 mice in each group), housed in sterile plastic package isolators and fed a sterilized normal diet. After 2 weeks of acclimation, the germ-free mice were orally gavaged with faecal suspensions from mice fed a HFD or subjected to LDR + HFD feeding. The mice were killed to collect tissues for further detection of various parameters at the end of the experiment. After all these experiments, the mice were fasted overnight before being euthanized. Blood samples were collected and kept at room temperature for 1 h to ensure complete clotting before centrifugation at 4 °C and 5000 rpm for 5 min to obtain serum samples. Faeces and tissues such as liver tissue, BAT, hippocampal tissue, and intestinal tissue were carefully collected, flash-frozen in liquid nitrogen and then stored at −80 °C until assessment.

### Analysis of metabolic parameters

For the OGTT, mice were fasted overnight prior to being treated with 1 g/kg glucose orally. Plasma samples were collected from the tail vein at 0, 5, 15, 30, 60, 90 and 120 min to assess glucose levels (Sinocare glucometer; Sinocare China). The insulin concentration was analysed with an enzyme-linked immunosorbent assay (ELISA) kit (Xinle Bio Co., Ltd., Shanghai, China) according to the manufacturer’s instructions. The ITT was conducted in mice deprived of food for 5 h prior to intraperitoneal injection with 1 U/kg insulin. The HOMA-IR score was calculated using the following formula: [fasting insulin concentration (mU/L) × fasting glucose concentration (mg/dL) × 0.05551]/22.5. For physiological function-related parameter detection, blood samples were collected in heparin-coated tube via eye punctures and centrifuged, and then the plasma samples were stored at −80 °C. Plasma cholesterol, HDL, LDL, AST and ALT levels were measured with a biochemistry platform using an Olympus AU400 Chemistry Analyser provided by Central South University. For the intestinal permeability assay, mice were orally treated with 4 kDa FITC-labelled dextran, and fluorescence in the serum was measured at the indicated timepoints^71^.

### H&E immunohistochemistry (IHC) and IF staining

BAT, liver tissues, hippocampal tissues and colon tissues were embedded in paraffin for staining with H&E according to previous reports^72^. For IHC, the paraffin sections were placed in citrate buffer for antigen retrieval and blocked in 3% H_2_O_2_. After incubation with the appropriate primary antibody and secondary antibody, the paraffin sections were blocked with DAB chromogen solution and HRP substrate solution. For IF staining, tissues were fixed with 4% paraformaldehyde at room temperature and permeabilized with 0.05% Triton X-100. Then, the tissues were stained with primary antibodies against the following proteins overnight at 4 °C: Occludin, ZO-1, E-cadherin, β-catenin and γ-H2AX. After washing with PBS 3 times, the cells were stained with secondary antibodies. The nuclei were stained with DAPI (Sigma). Images were acquired using a fluorescence confocal microscope (Nikon TI2-E, Crest Optics, X-Light V3, Italy).

### Electron microscopy for structural analysis of the gut, hippocampus and pancreas

TEM analysis was performed after collection of the gut, hippocampal and pancreatic tissues by Shiyanjia Lab (www.shiyanjia.com). The tissues were split and treated in a cold fixative solution composed of 2.5% glutaraldehyde at 4 °C for 4 h. After washing with PBS, the specimens were post-fixed in 1% OsO4 at 4 °C for 1 h and washed again with PBS. A graded series of ethanol solutions was used for further dehydration, and the specimens were transferred to be incubated. TEM was performed with a JEM-2100F at 80 kV, and images were acquired using a side-inserted BioScan camera. In addition, PA concentration in the study was detected by Shiyanjia Lab through HPLC (High performance liquid chromatography) analytical method according to the previous reported instructions^73^.

### Western blot analysis and quantitative real-time PCR (qRT-PCR)

Protein and mRNA expression was assessed using Western blotting and qRT-PCR methods. For Western blotting, proteins from gut tissue, liver tissue, hippocampal tissue and BAT were extracted using protein extraction reagent (Thermo Scientific, 78501). The total proteins were separated by SDS-polyacrylamide gel electrophoresis (SDS-PAGE) and transferred to a polyvinylidene fluoride (PVDF) membrane using a wet transfer apparatus (Bio-Rad PowerPac™ Basic, Bio-Rad Mini-Protean Tetra System 10025025 Rev A 12-06250312) as described in our previous reports^12,74^. ImageJ was used for band densitometry analysis. For RNA extraction from frozen tissues, TRIzol reagent (Jingcai Bio., Xi’an, Shanxi, China) was used. The relative expression of mRNA was quantified using SYBR Green dye (TB Green Premix Ex Taq II). Specific primers were designed by Green Pharma, Shanghai, China. QRT-PCR was performed with the following program: 95 °C for 10 min, 95 °C for 15 s, and 60 °C for 1 min in 40 cycles. The program was performed in a CFX96 Touch apparatus (Bio-Rad). The relative expression was calculated using the 2-ΔΔCT method.

### 16S rRNA microbiome sequencing

Faecal samples were collected based on the experimental design. The faecal samples were shipped to BioTREE Company, Shanghai, China, for detection using an Illumina NovaSeq. Total cellular DNA was extracted with an E.Z.N.A. Stool DNA Kit (Omega) based on the manufacturer’s instructions. The bacterial hypervariable V3-V4 region of 16 S rRNA was amplified using the primers 341_F:5′-CCTACGGGNGGCWGCAG-3′ and 802_R:5′-TACNVGGGTATCTAATCC-3′ according to our previously published study^13^. The library, which was constructed using a TruSeq® kit, was quantified with a Qubit instrument and qRT-PCR. After the library was qualified, a NovaSeq 6000 platform was used for sequencing. According to the barcode sequences and the PCR amplification primer sequences, the data for the different samples were separated. After the barcode and primer sequences were removed, FLASH (v1.2.7) was used (http://ccb.jhu.edu/software/FLASH/). The reads in each sample were spliced, and the spliced sequence was considered the raw tag. The raw tags obtained by splicing were filtered strictly to obtain high-quality tags. The QIIME pipeline (v1.9.1, http://qiime.org/scripts/split_ libraries) was used for tag quality control with FASTQ files. Briefly, for tag trimming, raw tags with continuous low qualityscores (default quality threshold: ≤19) were first cut from the first low-quality base site when the base number reached the set length (default length value: 3). Next, for tag length filtering, tags with continuous high-quality bases with lengths less than 75% of the tag length were further filtered out from the tags that remained after trimming. After the above processing steps, chimaeric sequences were removed from the tags. The tag sequences were compared to a species annotation database to detect chimerism (https://github.com/torognes/vsearch/), and the chimaeric sequences were removed. Finally, the clean tags were obtained. The UniFrac distance was calculated with QIIME software (version 1.9.1), and an unweighted pair group method with arithmetic mean (UPGMA) sample clustering tree was constructed. Principal component analysis (PCA), principal coordinate analysis (PCoA) and non-metric multi-dimensional scaling (NMDS) analysis were performed with R software (version 2.15.3). PCA was performed with R software’s ADE4 package and ggplot2 package; PCoA analysis was performed with R software’s WGCNA, stats and ggplot2 packages; and NMDS analysis was performed with R software’s vegan package. R software was used to analyse the differences between groups for beta diversity indices, and parametric tests and nonparametric tests were carried out. If there were only two groups, t-tests and Wilcoxon tests were used. If there were more than two groups, Tukey’s test and Wilcoxon test of theagricolae package were used.

### Untargeted metabolomics

Faecal and plasma samples were collected after mice were killed and stored at −80 °C until analysis. Analysis was carried out using ultrahigh-performance liquid tandem chromatography quadrupole time-of-flight mass spectrometry (UHPLC-QTOFMS) by BioTREE Company, Shanghai, China. During detection, both positive ion (POS) mode and negative ion (NEG) mode were used. Then, the missing values were filled with half of the minimum value. Additionally, the internal standard normalization method was employed in this data analysis. The final dataset containing the peak number, sample name and normalized peak area information was imported into the SIMCA15.0.2 software package (Sartorius Stedim Data Analytics AB, Umea, Sweden) for multivariate analysis. The data were scaled and logarithmically transformed to minimize the impacts of both noise and high variance of the variables. After these transformations, PCA, an unsupervised analysis that reduces the dimensionality of the data, was carried out to visualize the distribution and grouping of the samples. The 95% confidence interval in the PCA score plot was used as the threshold to identify potential outliers in the dataset. To visualize group separation and find changed metabolites, supervised orthogonal projections to latent structures-discriminant analysis (OPLS-DA) was applied. Then, a 7-fold cross validation was performed to calculate the values of R2 and Q2.TheR2 value indicates how well the variation of a variable is explained, and the Q2 value indicates how well a variable can be predicted. To assess the robustness and predictive ability of the OPLS-DA model, 200 permutations were further conducted. Afterward, the R2 and Q2 intercept values were obtained. Here, the intercept value of Q2 represents the robustness of the model, the risk of overfitting and the reliability of the model (smaller value is better). Furthermore, the variable importance in the projection (VIP) value of the first principal component in OPLS-DA was obtained. This value summarizes the contribution of each variable to the model. The metabolites with VIP > 1 and *p* < 0.05 (Student’s t-test) were considered significantly changed metabolites. In addition, commercial databases, including the KEGG (http://www.genome.jp/kegg/) and Metabo Analyst (http://www.metaboanalyst.ca/) databases, were used for pathway enrichment analysis.

### Statistics and reproducibility

Gut microbiota abundance, relative metabolite levels, relative protein expression and mRNA expression are presented as the means ± SDs. Significant differences between mean results were analysed by Student’s t-test and one-way ANOVA, but the results of the antibiotic, PA and probiotic intervention experiments were analysed by two-way ANOVA with LDR, LDR + HFD + antibiotics, PA and probiotics as factors. The statistical figures were drawn and multiple comparisons via Tukey’s test were carried out with GraphPad Prism 8.0 software. Image J was used for band densitometry analysis. A value of *p* < 0.05 was considered to indicate a significant difference between means.

### Reporting summary

Further information on research design is available in the Nature Research Reporting Summary linked to this article.

## Supplementary information

Supplementary Information

Description of Additional Supplementary Files

Supplementary Data 1

Reporting Summary

## Data Availability

The source data for all the graphs prepared for the manuscript is available as Supplementary Data 1. Uncropped blots used for preparing figures in the manuscript are available as Supplementary Fig. 8. Due to the guideline of the Journal that all the WB results must be accompanied by uncropped gels, we then re-conducted experiment and the uncropped gels of WB are illustrated in the Supplementary file of Supplementary Fig. 8. Supplementary Fig. 9 and Supplementary Fig. 10 were submit to the Journal at the first time without available of uncropped gels. The raw data of fecal 16rs are available also in the supplementary data. The raw data of fecal 16rs have been deposited at BioProject and is accessible via accession number PRJNA834924. All other data are available from the corresponding authors upon reasonable request.
