# Supplementary material for: Low-dose radiation exaggerates HFD-induced metabolic dysfunction by gut microbiota through PA-PYCR1 axis
Source: Commun Biol. 2022 Sep 10;5:945. doi: 10.1038/s42003-022-03929-1 (PMC9464247; doi:10.1038/s42003-022-03929-1)
Supplement: Supplementary file 1 — Supplementary Information [file 42003_2022_3929_MOESM1_ESM.pdf]

a

	Con	Radiotherapy	T2D	Radiotherapy+ T2D
Gender				
Male	3	2	4	3
Female	2	3	1	2
Age(Year)				
>60	2	3	3	2
<60	3	2	2	3
BMI	21.63±3.78	22.17±4.15	26.19±5.1 9	27.13±1.09
Fasting Glucose(mm ol/L)	4.98±0.96	5.71±1.44	8.05±1.37	9.06±2.05
HbA1c(%)	5.12±1.27	5.64±0.82	7.64±2.06	8.43±1.91

b

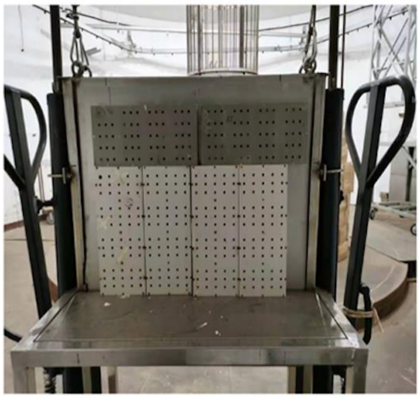

c

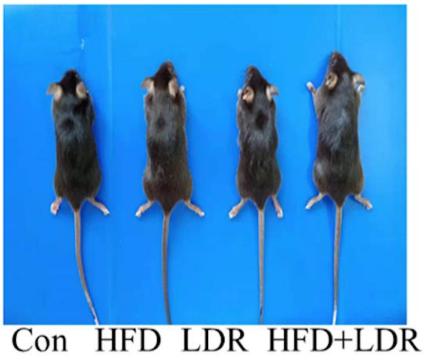

d

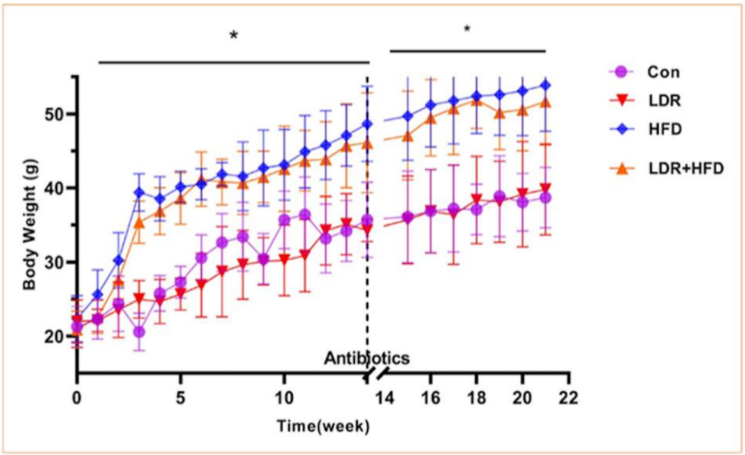

Supplementary Figure 1. Body weight alterations among the mice. a. Baseline information for the different clinical cases. b. Radiation device used for mice LDR treatment. c. Representative photos of mice from the four groups. d. Body weight alterations in mice over 21 weeks among the groups.

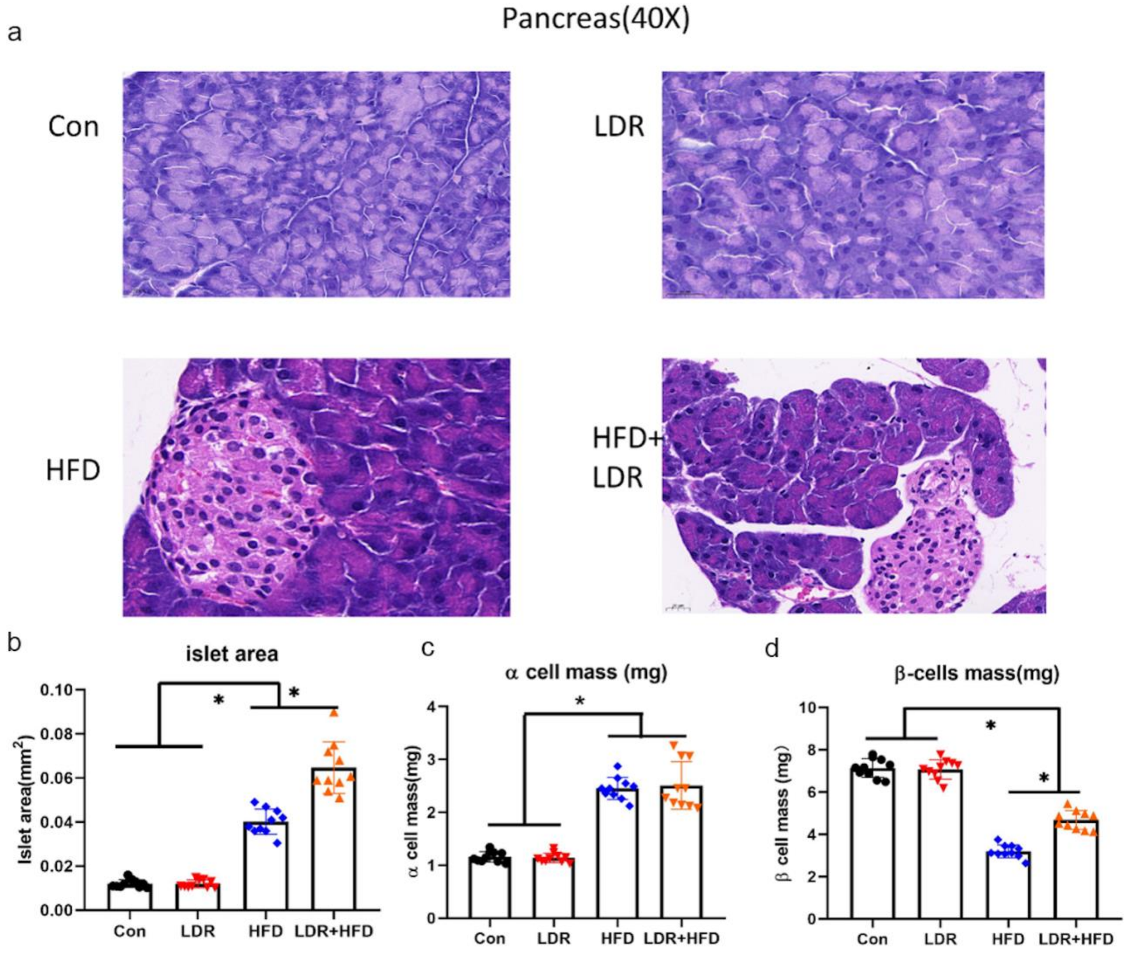

Supplementary Figure 2. Pancreas alterations in mice. a. Representative pictures of pancreatic tissue stained with H&E (40X). b. Islet areas in mice from the different groups. c. αCell masses in mice from the different groups. d. βCell masses in mice from the different groups. The data are the means ± SDs. *p<0.05 indicates a significant difference.

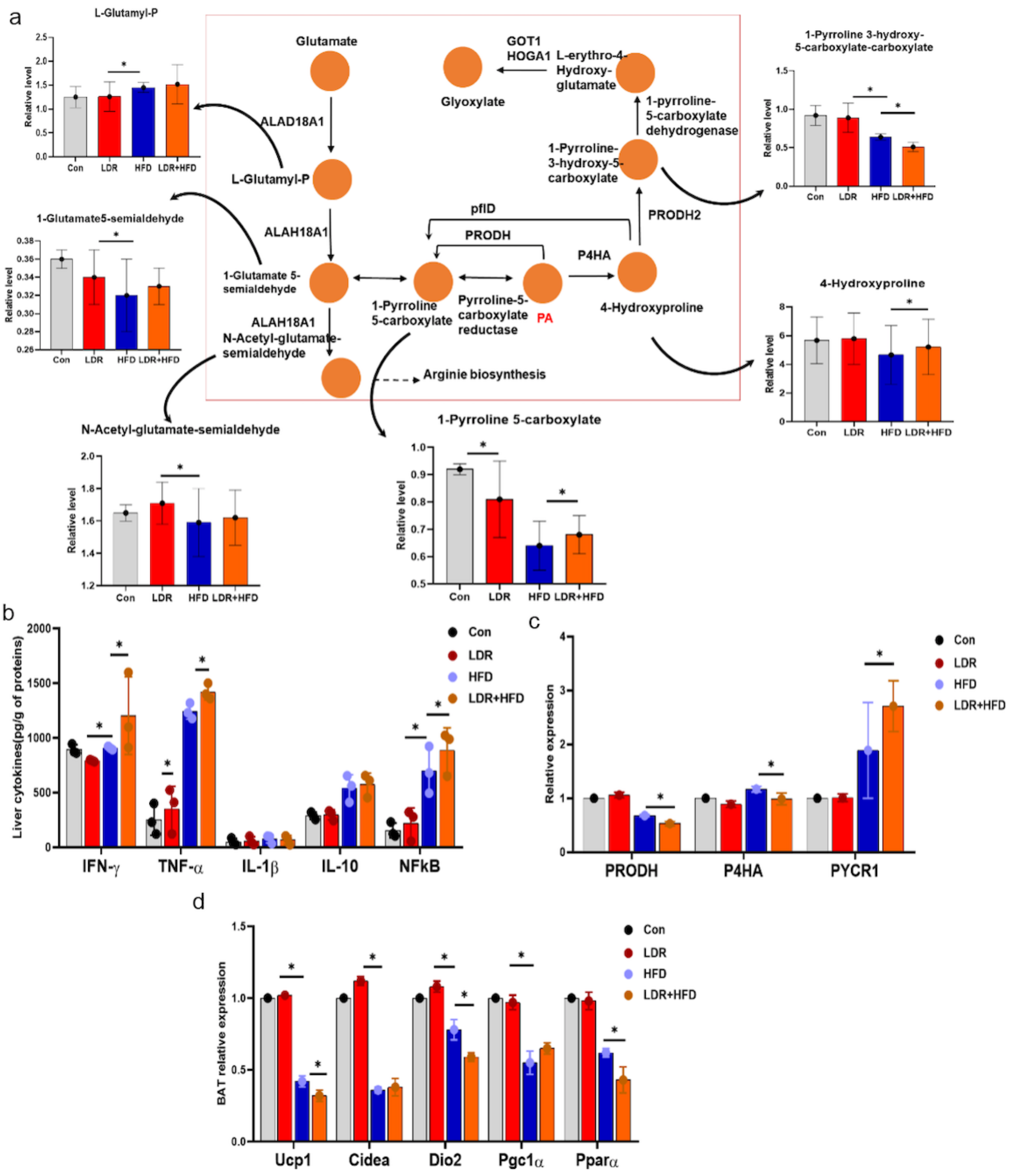

Supplementary Figure 3. LDR disturbs HFD-induced PA metabolism. a. PA metabolic network integrated with detected metabolites. Levels of the identified metabolites L-glutamyl-P, 1-glutamate-5-semialdehyde, N-acetyl-glutamate-semialdehyde, 1-pyrroline5-carboxylate, 4-hydroxyproline and 1-pyrroline 3-hydroxy-5-carboxylate-carboxylate among the Con, LDR, HFD and LDR+HFD groups. b. Cytokine levels in liver tissues. c. PA synthesis and decomposition-related gene expression in liver tissues (n=3 per group). d. BAT biomarker expression in BAT. The data are the means ± SDs. The Mann-Whitney test or two-tailed unpaired Student's t-test was used for

statistical analyses. *p<0.05 indicates a significant difference.

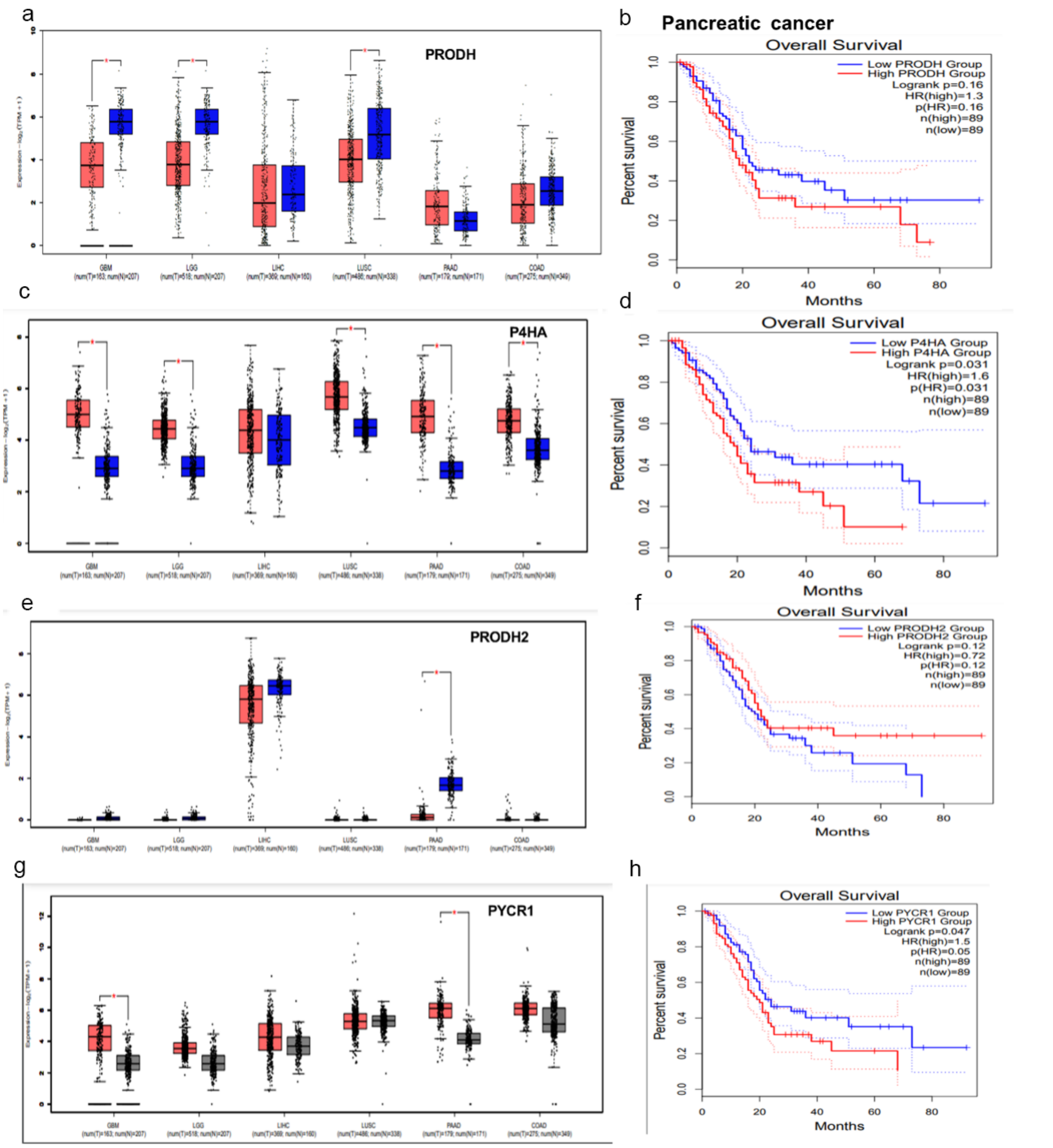

Supplementary Figure 4. PA pathway regulation-related gene expression in various cancer tissues and overall survival rate analysis. a. Comparison of PRODH expression in various cancer tissues and normal tissues using the online GEPIA2 database (http://gepia2.cancer-pku.cn/#index). b. Overall survival rates of pancreatic cancer patients with low PRODH expression and high PRODH expression from the online GEPIA2 database (http://gepia2.cancer-pku.cn/#index). c. Comparison of P4HA

expression in various cancer tissues and normal tissues using the online GEPIA2 database (http://gepia2.cancer-pku.cn/#index). d. Overall survival rates of pancreatic cancer patients with low P4HA expression and high P4HA expression from the online GEPIA2 database (http://gepia2.cancer-pku.cn/#index). e. Comparison of PRODH2 expression in various cancer tissues and normal tissues using the online GEPIA2 database (http://gepia2.cancer-pku.cn/#index). f. Overall survival rates of pancreatic cancer patients with low PRODH2 expression and high P4HA expression from the online GEPIA2 database (http://gepia2.cancer-pku.cn/#index). g. Comparison of PYCR1 expression in various cancer tissues and normal tissues using the online GEPIA2 database (http://gepia2.cancer-pku.cn/#index). h. Overall survival rates of pancreatic cancer patients with low PYCR1 expression and high PYCR1 expression from the online GEPIA2 database(http://gepia2.cancer-pku.cn/#index).The abbreviations are as follows: LGG, brain lower-grade glioma; LIHC, liver hepatocellular carcinoma; LUSC, lung squamous cell carcinoma; PAAD, pancreatic adenocarcinoma; GBM, glioblastoma multiforme; COAD, colon adenocarcinoma.

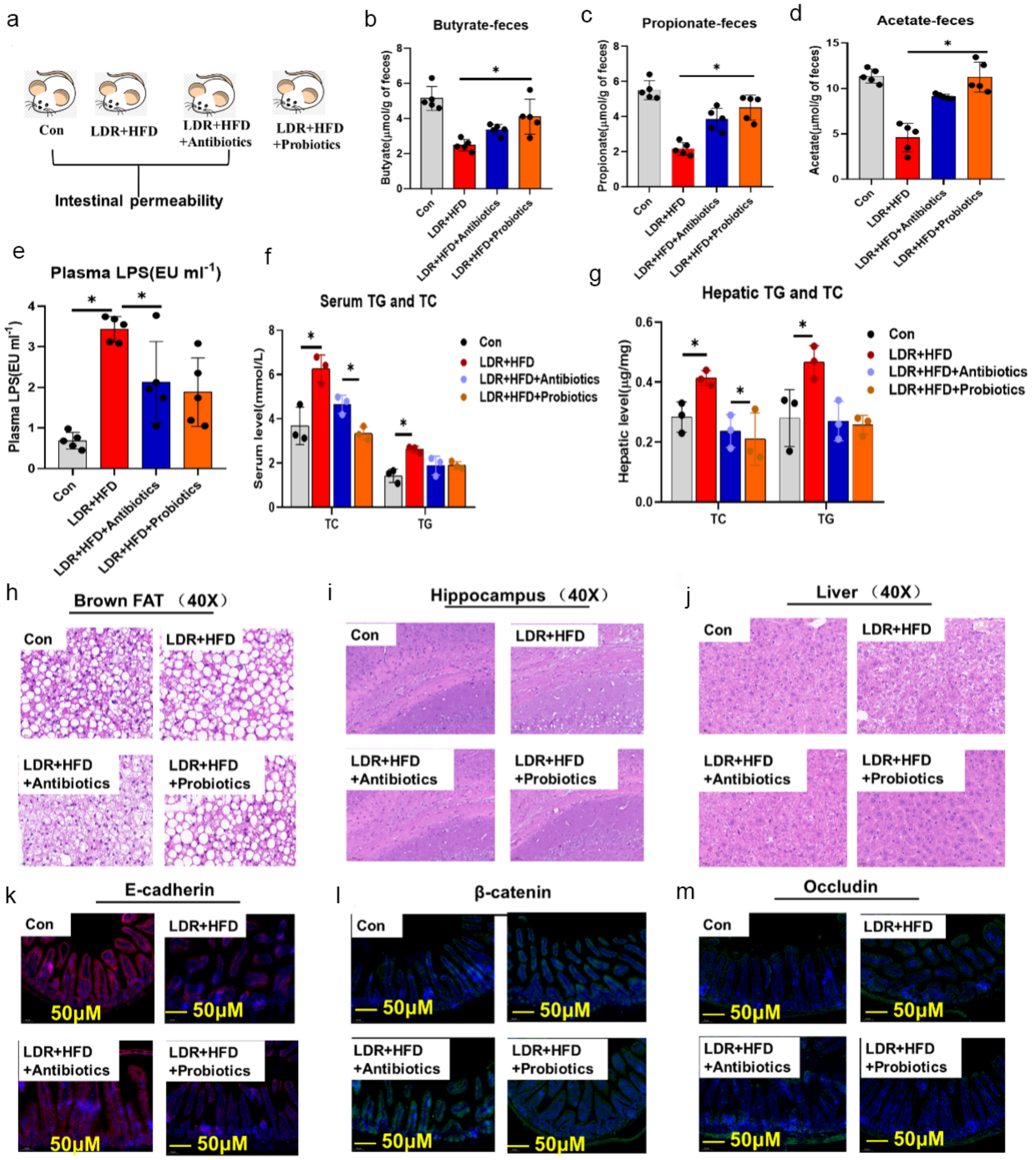

Supplementary Figure 5. Probiotic intervention reverses LDR+HFD-induced intestinal impairments. Mice were divided into four groups: the Con group, the LDR+HFD group, the LDR+HFD+antibiotics (cocktail) group, and the LDR+HFD+probiotics (*Lactobacillus rhamnosus* and *Lactobacillus reuteri* complex) group. a. Timeline of the treatment of the mice with LDR+HFD, LDR+HFD+antibiotics, and LDR+HFD+probiotics. b. Butyrate concentrations in faeces. c. Propionate concentrations in faeces. d. Acetate concentrations in faeces. e. Plasma LPS levels. f. Serum TG and TC levels. g. Hepatic TG and TC levels. h-j.H&E staining of representative BAT (h), hippocampal (i) and liver (j)

tissues(40X). k-m. IF detection of E-cadherin (k), β-catenin (l) and Occludin (m) in mouse intestinal tissues. Scale bar: 50 μm. The data are the means ± SDs. The Mann-Whitney test or two-tailed unpaired Student's t-test was used for statistical analyses. *$p<0.05$ indicates a significant difference.

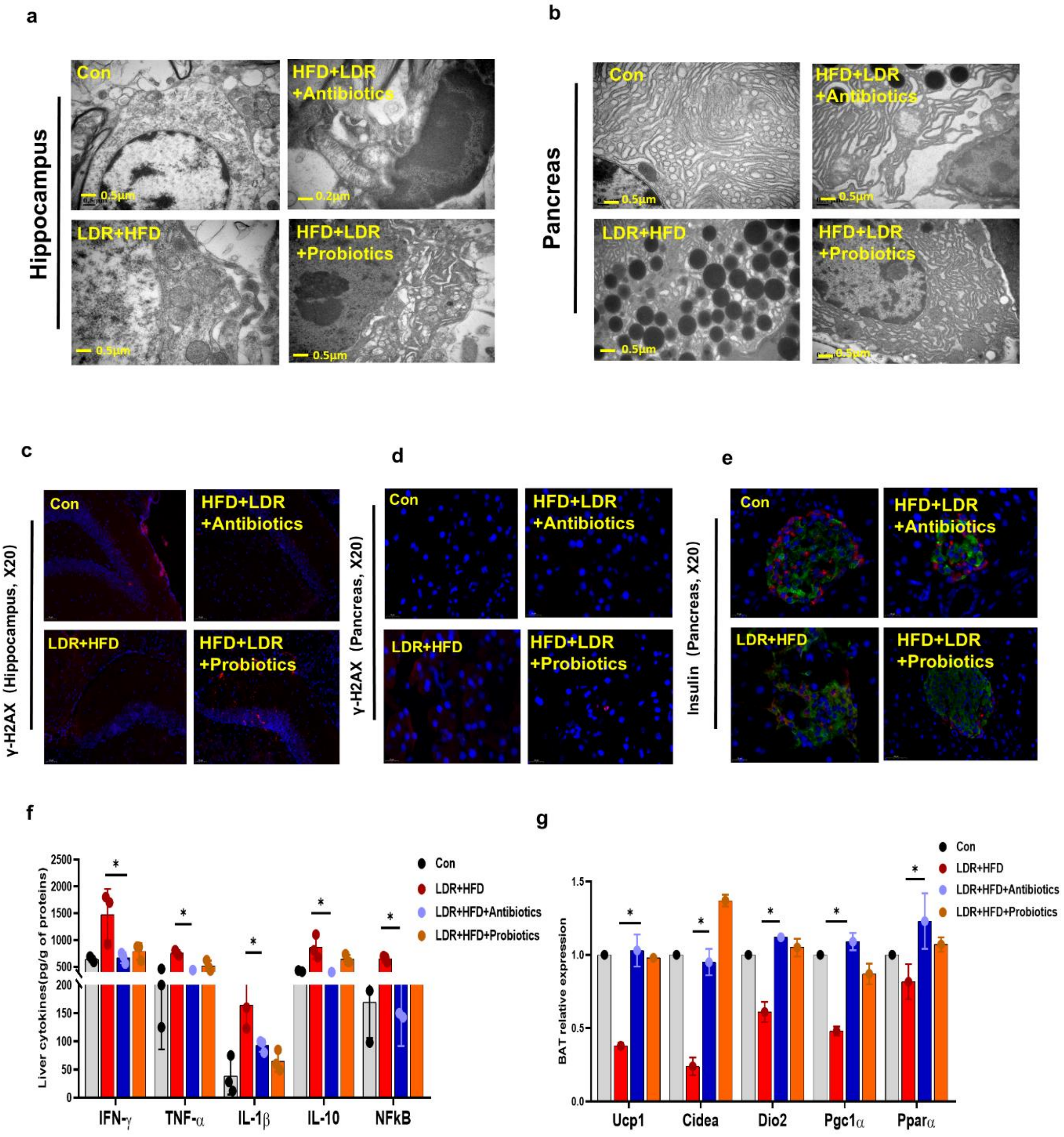

Supplementary Figure 6. Probiotic intervention reverses LDR+HFD-induced metabolic impairments. Mice were divided into four groups: the Con group, the LDR+HFD group, the LDR+HFD+antibiotics (cocktail) group, and the LDR+HFD+probiotics (*Lactobacillus rhamnosus* and *Lactobacillus*

*reuteri*complex) groups. a-b. Representative electron microscopy images of the hippocampus (a) and pancreas (b) among the four groups. Scar bar=0.5μm. c-e. IF detection ofγ-H2Ax in hippocampal tissues (c), γ-H2Ax inpancreas tissues(d) and insulin levels (e). 20X. f. Liver cytokine levels in the four groups. g. BAT-related biomarker expression in the four groups. The data are the means ± SDs. The Mann-Whitney test or two-tailed unpaired Student’s t-test was used for statistical analyses. *p<0.05 indicates a significant difference.

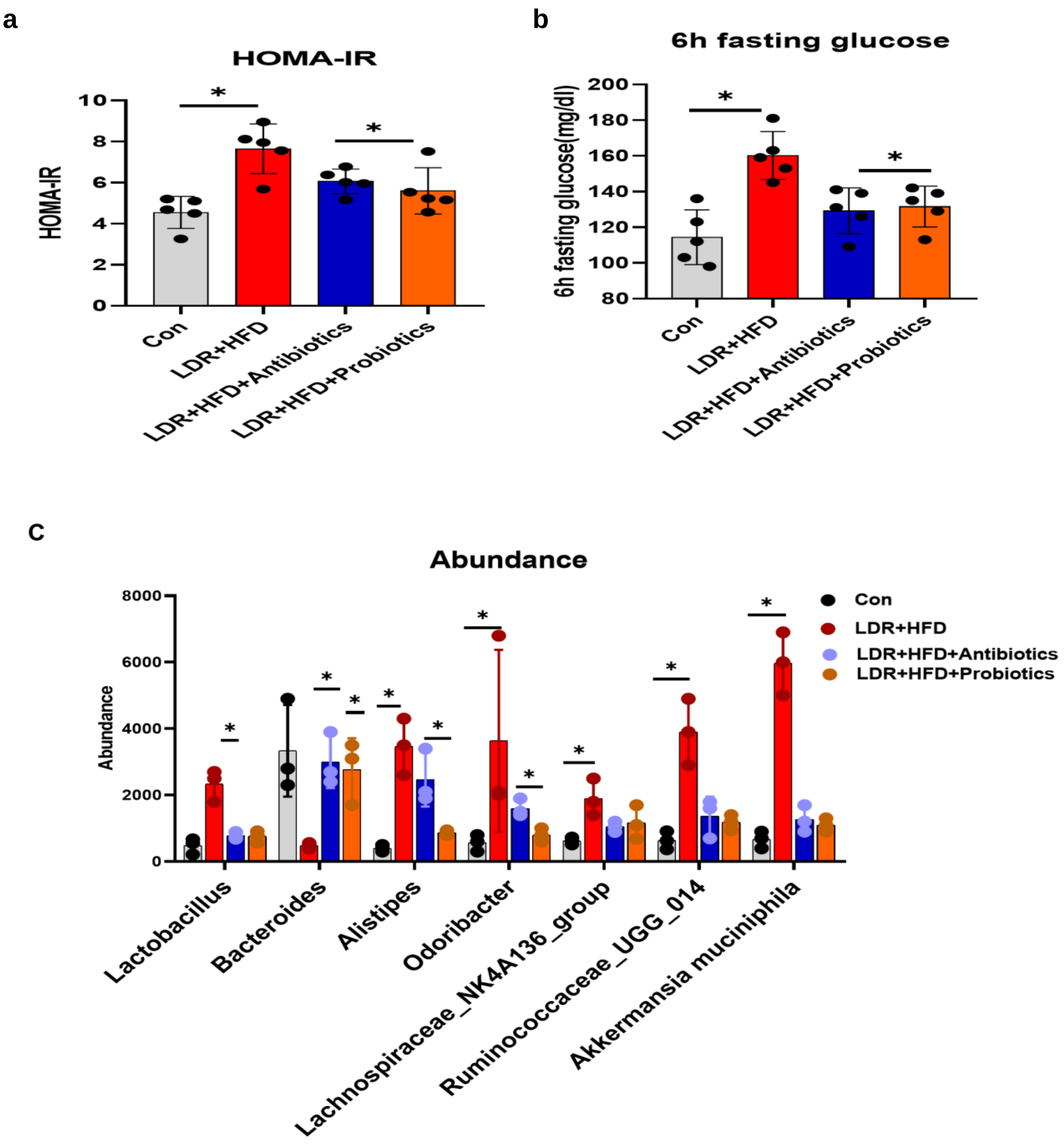

Supplementary Figure 7. Probiotic intervention reversed LDR+HFD-induced insulin resistance. Mice

were divided into four groups: the Con group, the LDR+HFD group, the LDR+HFD+antibiotics (cocktail) group, and the LDR+HFD+probiotics (*Lactobacillus rhamnosus*and *Lactobacillus reuteri* complex) group. a. HOMA-IR values for the four groups. b. Fasting glucose levels for the four groups. c. Abundance of gut microbes in faeces among the four mouse groups. The data are the means ± SDs. TheMann-Whitney test or two-tailed unpaired Student's t-test was used for statistical analyses. *$p<0.05$ indicates a significant difference.

Fig 7b

p-IRS2

190kDa-

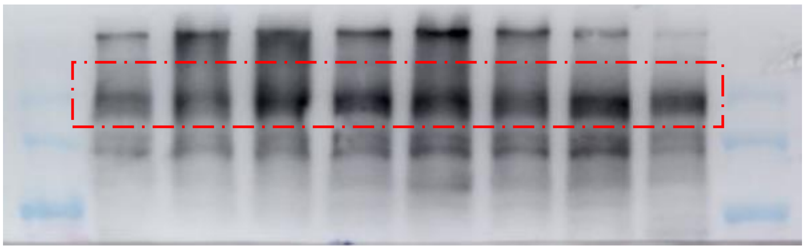

IRS2

190kDa-

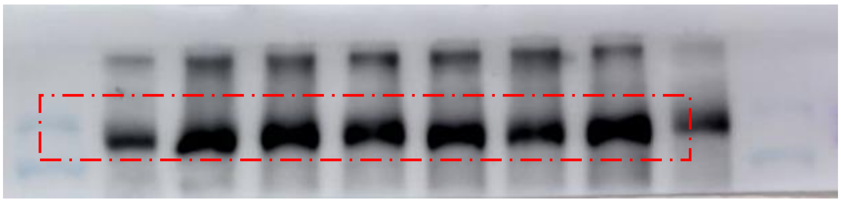

GAPDH

32kDa-

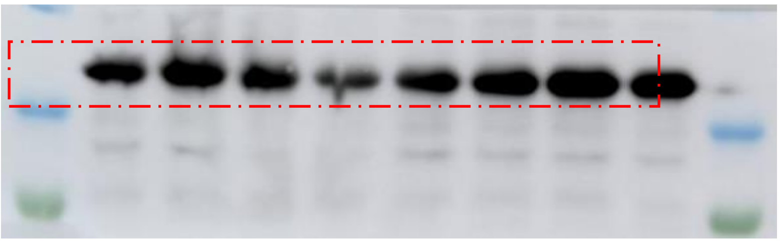

Fig 7c

p-IRS1

190kDa-

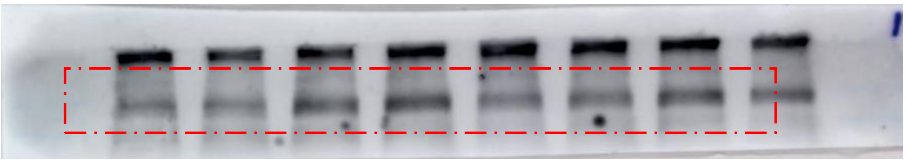

IRS1

190kDa-

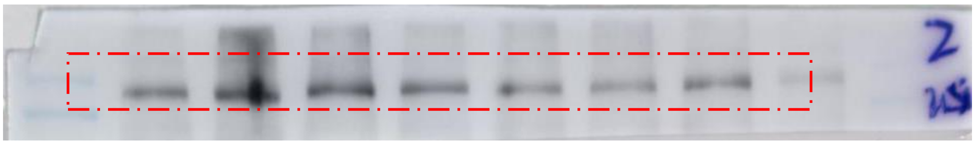

GAPDH

32kDa-

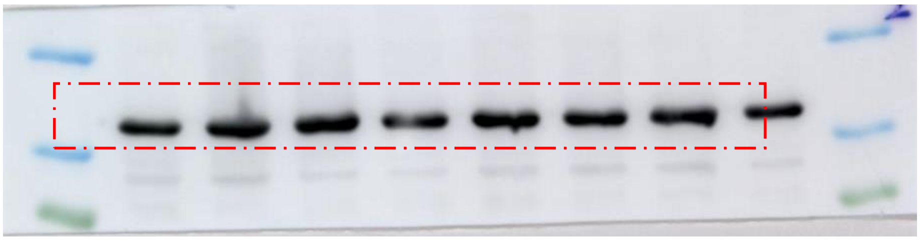

Fig 7d

p-PI3K

95kDa-

65kDa-

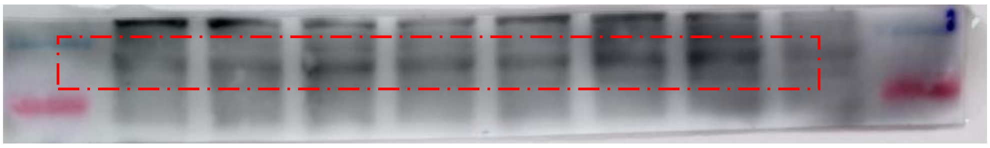

PI3K

95kDa-

65kDa-

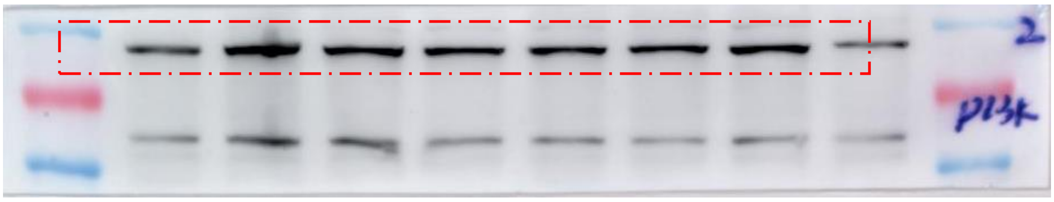

GAPDH

32kDa-

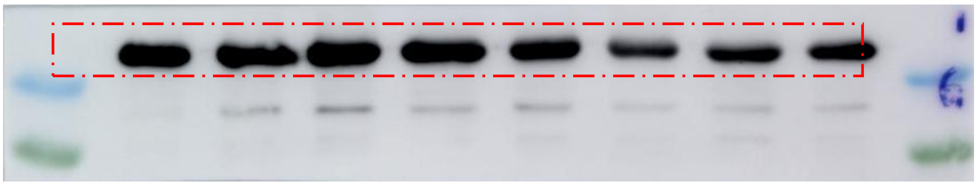

Fig 7e

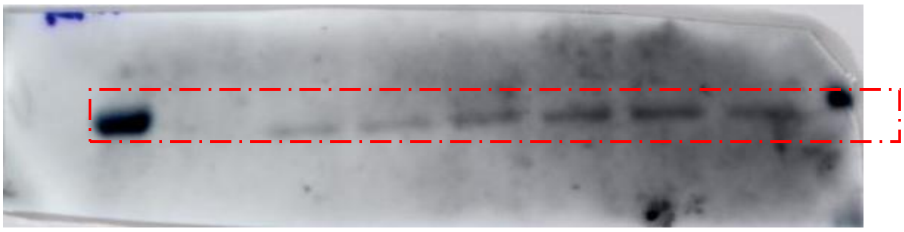

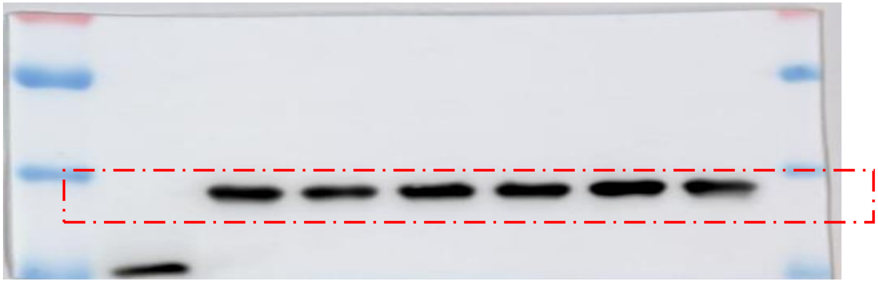

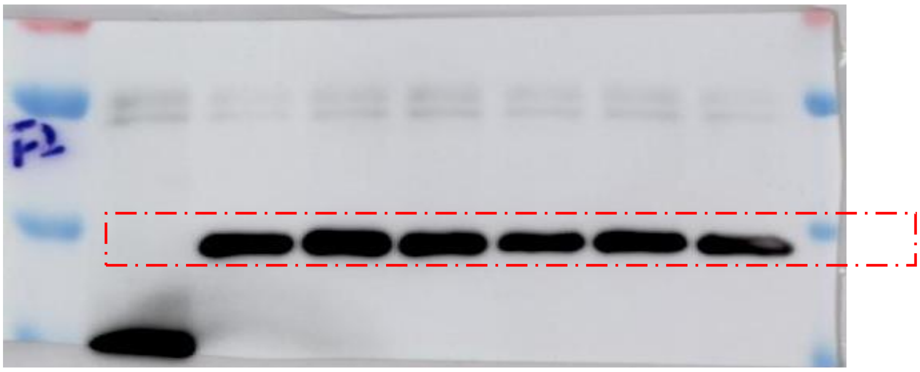

Fig 7f

AKT

65kDa-
54kDa-

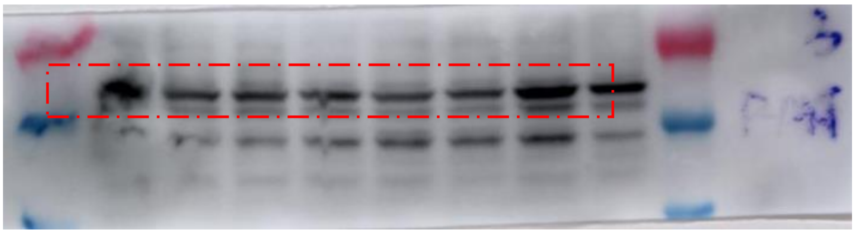

p-AKT

65kDa-
54kDa-

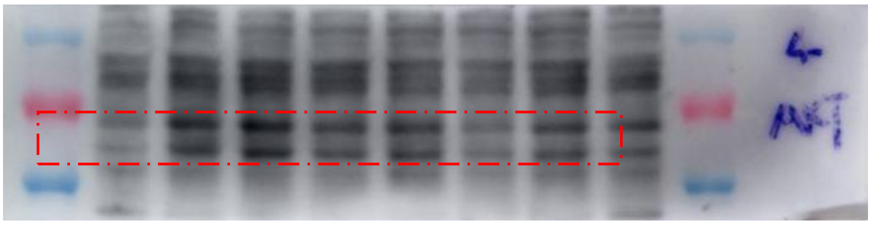

GAPDH

32kDa-

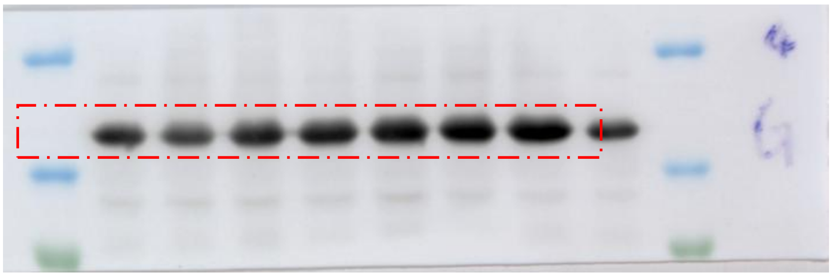

Fig 7g

PI3K

95kDa-
65kDa-

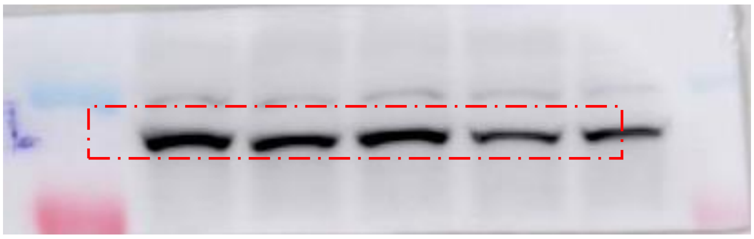

NFκB

65kDa-

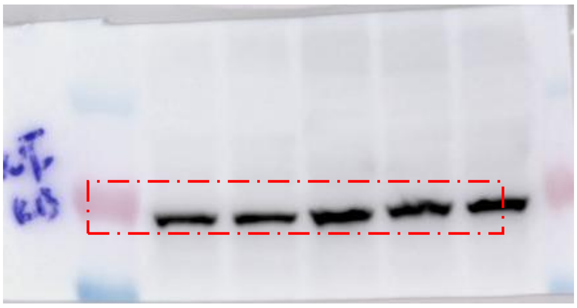

mTOR

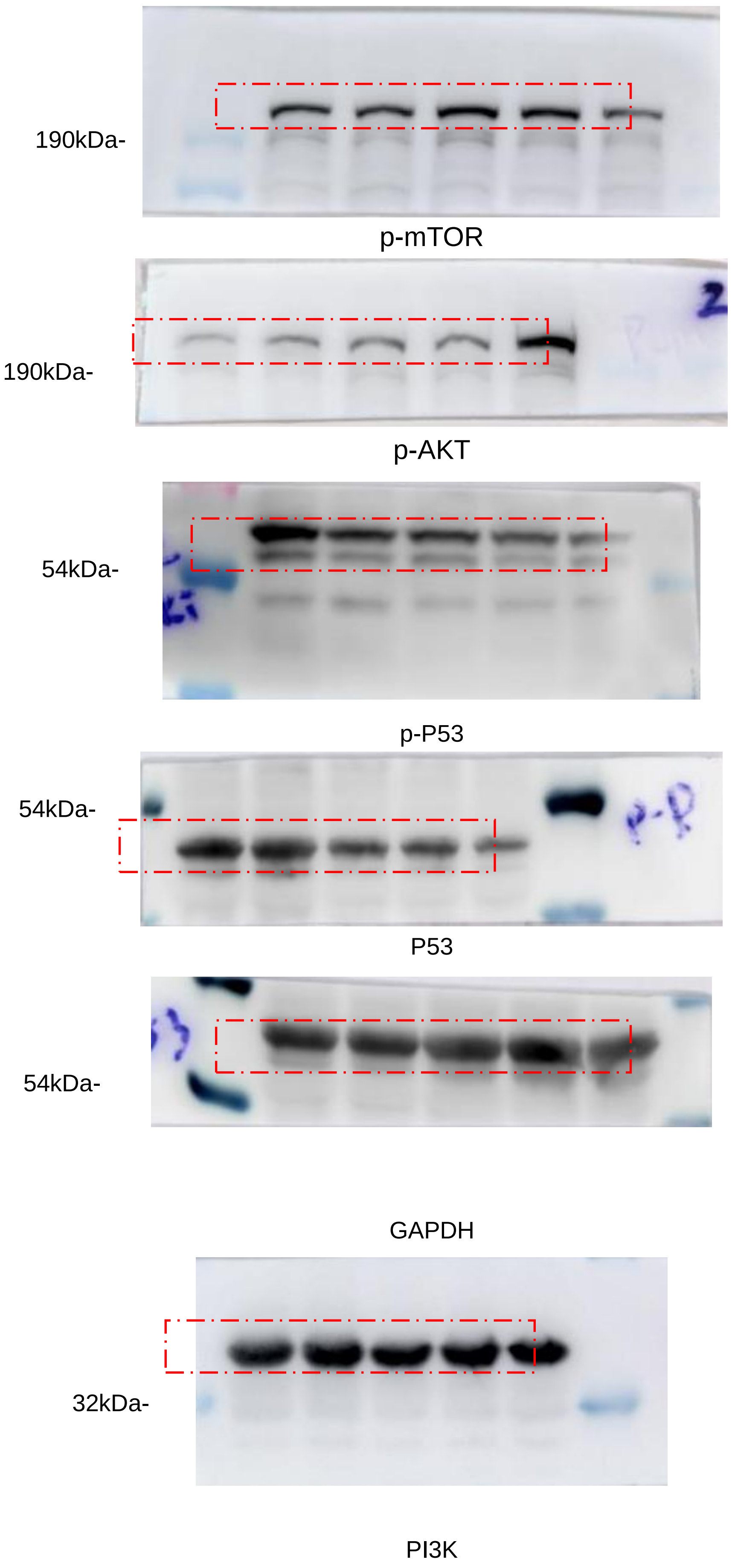
190kDa-
p-mTOR
190kDa-
p-AKT
54kDa-
p-P53
54kDa-
P53
54kDa-
GAPDH
32kDa-
PI3K

Fig 8c

95kDa-

65kDa-

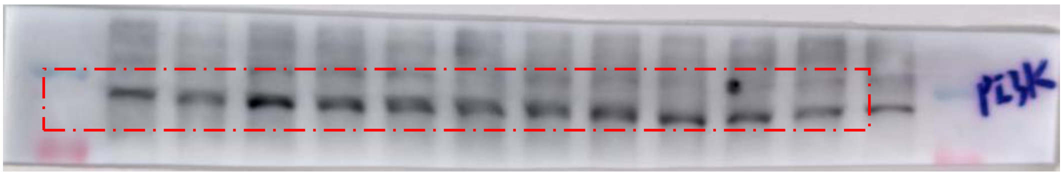

Akt

54kDa-

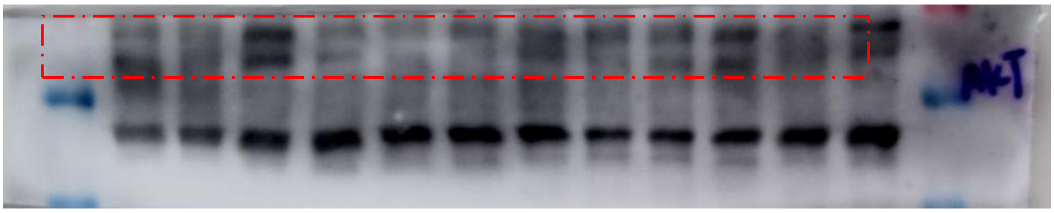

p-Akt

54kDa-

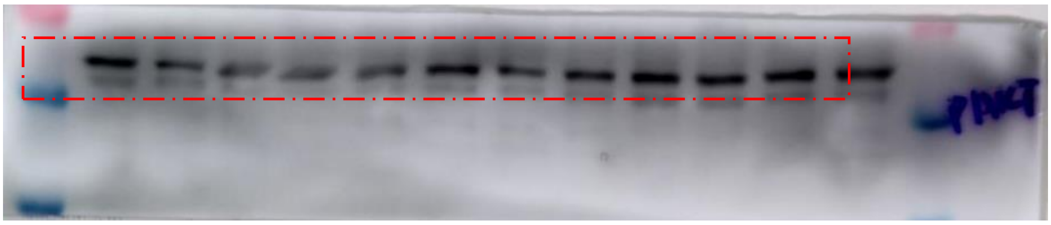

IRS-1

190kDa-

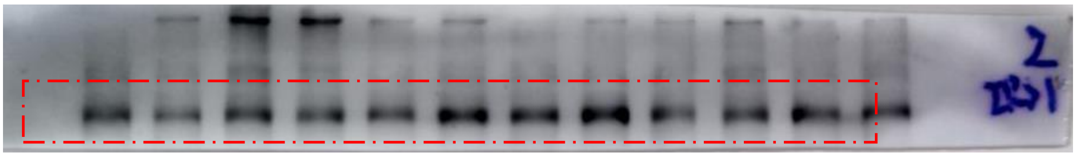

p-IRS-1

190kDa-

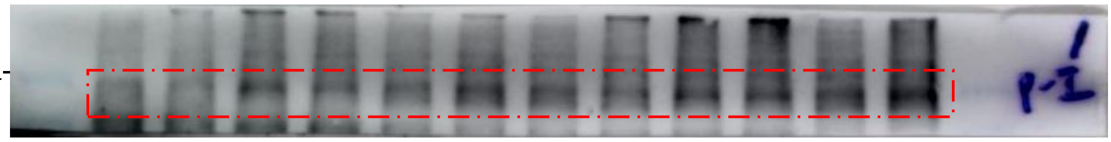

mTOR

190kDa-

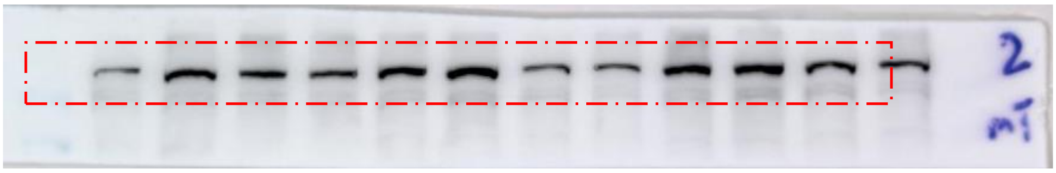

GAPDH

32kDa-

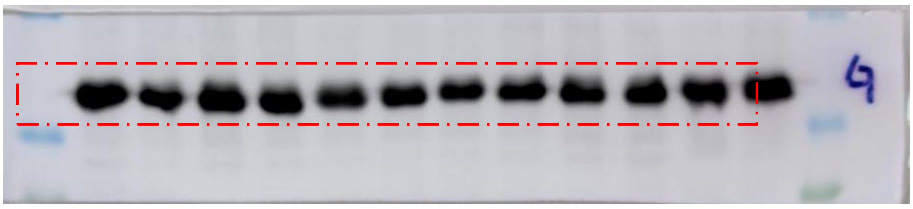

Fig 8e

p-IRS-1

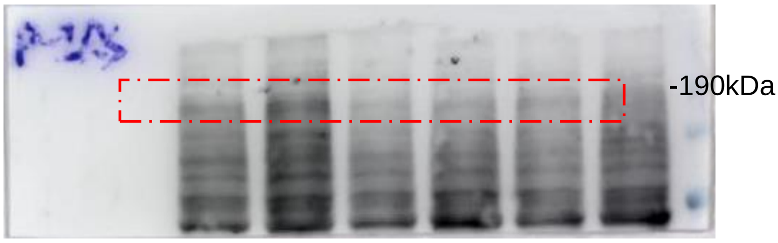
-190kDa

IRS-1

190kDa-

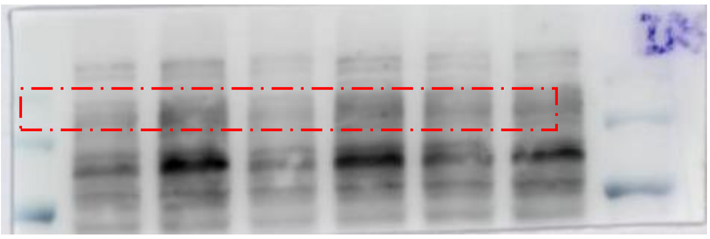

p-mTOR

190kDa-

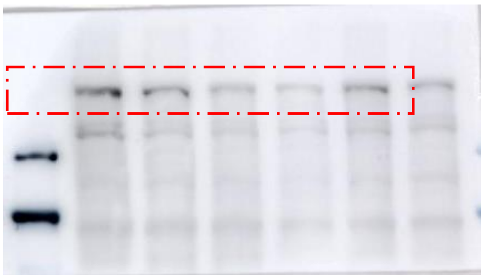

p-AKT

65kDa-

54kDa-

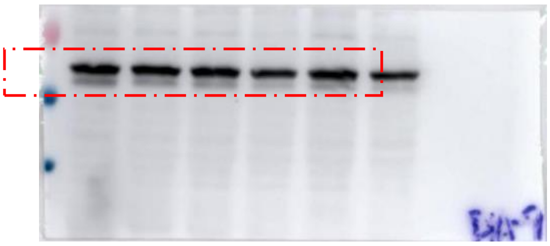

AKT

65kDa-

54kDa-

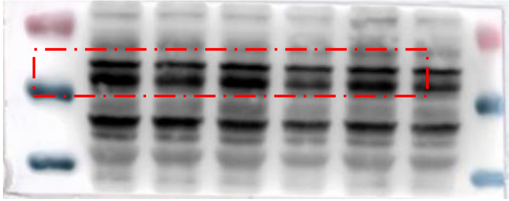

p-PI3K

95kDa-

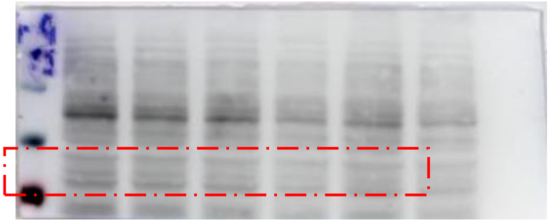

PI3K

95kDa-

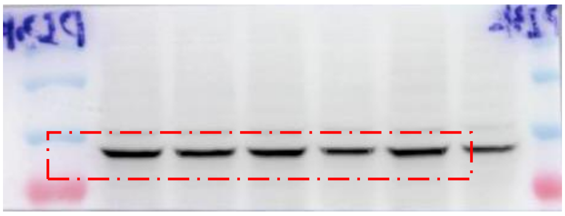

GAPDH

32kDa-

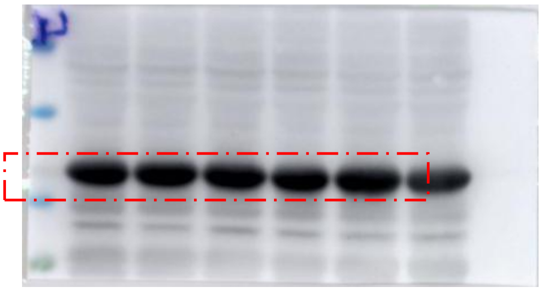

Supplementary Figure 8. For uncropped gels.

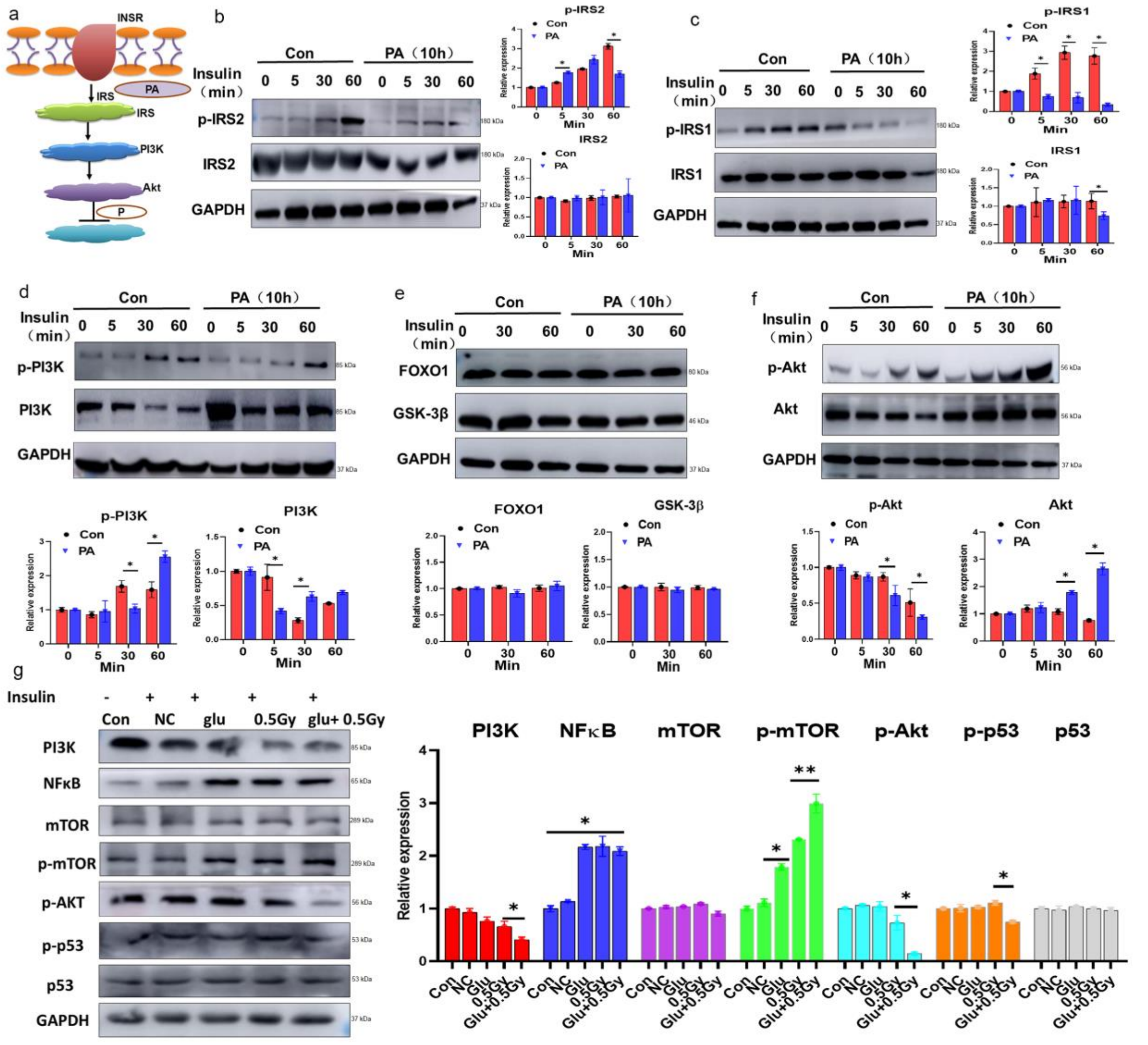

Supplementary Figure 9. The previous Western blot gels for the innate submission of Figure 7. Due to the guideline of Journal that all the WB results must be accompanied by uncropped gels, thus, these WB results have been repeated.

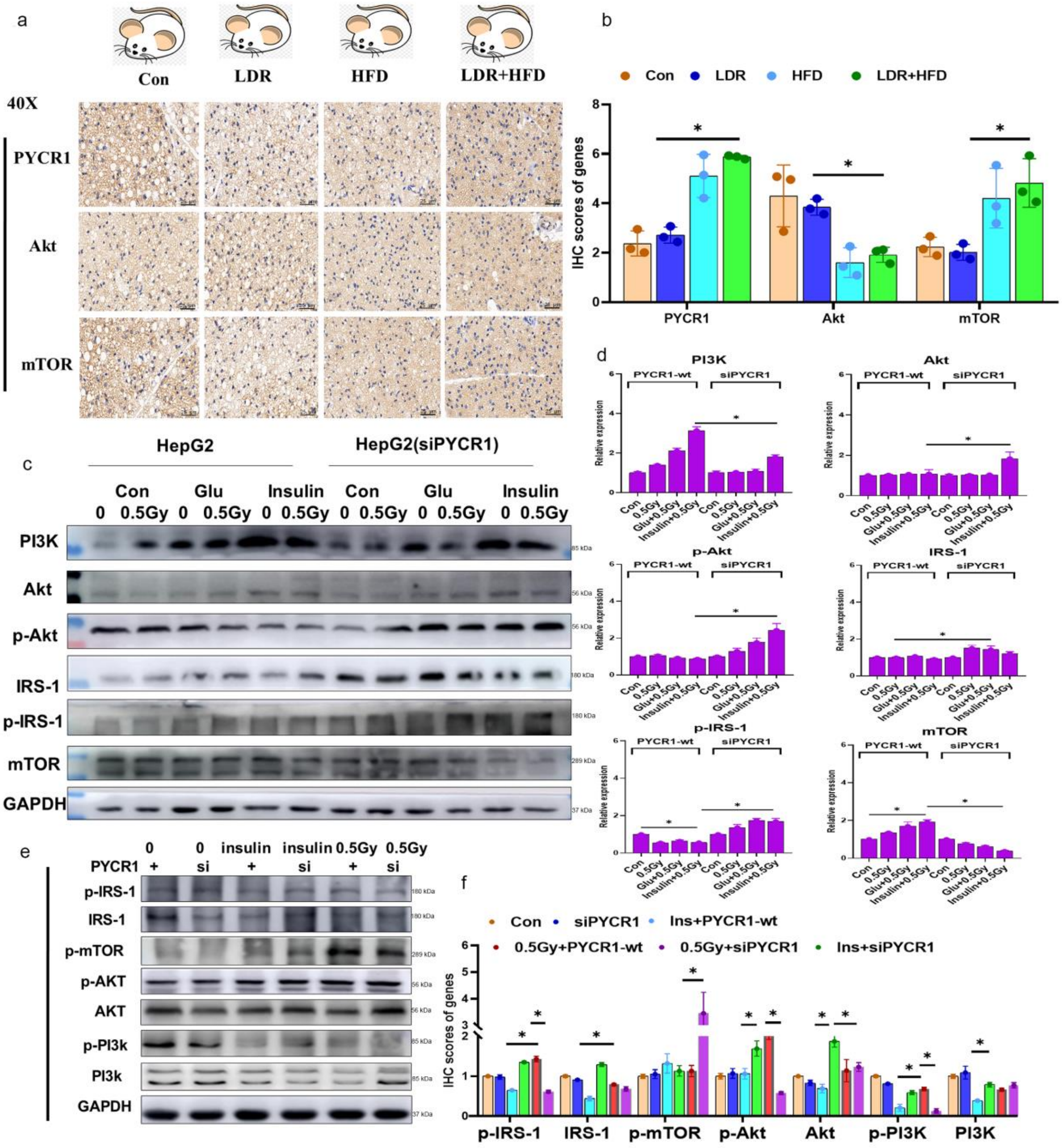

Supplementary Figure 10. The previous Western blot gels for the innate submission of Figure 8. Due to the guideline of Journal that all the WB results must be accompanied by uncropped gels, thus, these WB results have been repeated.

Supplementary table 1 | Comparative alterations of gut microbiota relative abundance among groups at 21 weeks in mice

Up (LDR+HFD vs. HFD)					Down(LDR+HFD vs. HFD)				
Relative abundance(Genus)					Relative abundance(Genus)				
**Taxonomy**	**Con**	**LDR**	**HFD**	**LDR+HFD**	**Taxonomy**	**Con**	**IR**	**HFD**	**LDR+HFD**
Alistipes	0.006032	0.003032	0.015076	0.076613	Akkermansiamuciniphila	0.02953	0.013156	0.019236	0.007117
Desulfovibrio	0.018631	0.01771	0.014636	0.195496	Bacteroides	0.317477	0.35372	0.017634	0.002494
Muribaculum	0.0017457	0.0019707	0.010759	0.093911	Prevotellaceae_UCG_001	0.44367	0.24886	0.034666	0.031403
Oscillibacter	0.012484	0.02384	0.013384	0.013633	Rikenellacear_RC9_gut_group	0.004355	0.004305	0.015159	0.003775
Alloprevotella	0.006603	0.017382	0.010705	0.011305	Butyric coccus	0.007519	0.002994	0.001676	0.001561
Intestinimonas	0.010116	0.003521	0.007188	0.011042	Anaeroplasma	0.00333	0.01153	0.00467	0.001045
Ruminococcaceae_UCG_014	0.005425	0.01248	0.012252	0.010071	Marvinbryantia	0.002182	0.001609	0.001609	0.000764
Blautia	0.003836	0.003785	0.07467	0.081237	Tyzzerella	0.000647	0.000659	0.000535	0.00048
Lachnoclostridium	0.002941	0.009776	0.003053	0.005318	Acetatifactor	0.000311	0.00056	0.000535	0.000423
Erysipelatoclostridium	0.007055	0.008054	0.004147	0.0053	Peptococcus	0.000071	0.000174	0.000228	0.000149
Rikenella	0.0017	0.00051	0.000477	0.00455	Harryflintia	0.000112	0.000091	0.000091	0.000057
Parabacteroides	0.003372	0.00776	0.002439	0.003701	Weissella	0.000004	0	0.000203	0.000025
Odoribacter	0.000755	0.000805	0.000722	0.003519	Gemella	0.000004	0.000012	0.000004	0.000021
Parasutterella	0.001597	0.001638	0.033061	0.386331	Candidatus_Stoquefichus	0.000008	0.000012	0.000037	0.000018
Ruminiclostridium					Parvibacter	0.000071	0.000033	0.000029	0.000014

Supplementary table 2 | Differentialalterations of plasma metabolites (*p*<0.05)in mice at 21 week

LDR+HFD vs. HFD						LDR vs. Con					
Up			Down			Up			Down		
Name	SC	FC	Name	SC	FC	Name	SC	FC	Name	SC	FC
Tridecanoic acid	LLM	479.80	Decanoylcarnitine	LLM	0.497304862	Nicotinamide N-oxide	OC	6.1232480	Histidinal	ONC	0.30472531
Heneicosanoic acid	LLM	15.578	4a-Hydroxytetrahydrobiopterin4A	OC	0.551010663	Koeniginequinone B	OC	1.8344312	Apigenin 7-sulfate	PPP	0.35852376
2-acetyl-1-alkyl-sn-glycero-3-phosphocholine2-	LLM	3.2136	L-Octanoylcarnitine	LLM	0.595171423	N-Acetylglutamic acid	OAD	1.5967982	Genistein	PPP	0.45044178
3-(3,4-Dihydroxy-5-methoxy)-2-propenoic acid3-	OAD	1.80732	L-Hexanoylcarnitine	LLM	0.627841431	PC(16:0/P-16:0)	LLM	1.4923108	Biochanin A	PPP	0.46304097
MethyImidazoleacetic acid	OC	1.48903	cis-5-Tetradecenoylcarnitine	LLM	0.667307112	4-Trimethylammoniobutanoic acid	LLM	1.4158858	Palmitoylethanolamide	OAD	0.53660155
Thymidine	NNA	1.353678463	L-Acetylcarnitine	LLM	0.668543891	PC(P-18:1(11Z)/22:6(4Z,7Z,10Z,13Z,16Z,19Z))	LLM	1.3544444	Kynurenic acid	OC	0.54046059
N-Acetylornithine	OAD	1.331460731	Phytosphingosine	ONC	0.682301446	N2-gamma-Glutamylglutamine	OAD	1.2115518	Dethiobiotin	LLM	0.50430544
Deoxyuridine	NNA	1.279841779	D-Alanine	OAD	0.694127498	Guanidoacetic acid	OAD	1.1517346	Phytosphingosine	ONC	0.50354129
Pyrrolidonecarboxylic acid	OAD	1.254312949	Adenosine 2'-phosphate	OOC	0.710332455	PE(20:5(5Z,8Z,11Z,14Z,17Z)/P-18:0)	LLM	1.2221623	4-Aminophenol	Benzenoids	0.49508081
Carboxytolbutamide	Benzenoids	1.180351521	Sphinganine	ONC	0.740426587				PC(15:0/14:1(9Z))	LLM	0.47498049

Elaidic carnitine	LLM	0.754290455		PC-M5'	OC	0.50079322
L-Palmitoylcarnitine	LLM	0.783212053		L-phenylalanyl-L-hydroxyproline	OAD	0.46644688
N-Ornithyl-L-taurine	OAD	0.790077469		Sphinganine	ONC	0.45629106
N-Acetyldopamine	Benzenoids	0.800976194		m-Aminobenzoic acid	Benzenoids	0.49368323
Isotheaflavin	PPP	0.808477863		Valyl-Valine	OAD	0.50166812
2,5-Dihydro-2,4-dimethyloxazole	OC	0.855950944		Prolylhydroxyproline	OAD	0.49595862
Butyramide	LLM	0.868692479		Proline betaine	OAD	0.44652675
2-Piperidinone	OC	0.876125309	OC	gamma-Glutamyltyrosine	OAD	0.49780815
				PC(18:4(6Z,9Z,12Z,15Z)/15:0)	LLM	0.46231357
				L-Kynurenine	OOC	0.47511501
				Sphingosine 1-phosphate	LLM	0.49148378

LLM: Lipids and lipid-like molecules; PPP: Phenylpropanoids and polyketides; OAD: Organic acids and derivatives; OOC: Organic oxygen compounds; ONC: Organic nitrogen compounds; NNA: Nucleosides, nucleotides, and analogues; OC: Organoheterocyclic compounds; FC: fold change; SC:superclass

Supplementary table 3 | Differentialalterationsoffecal metabolites ($p<0.05$)among groups in mice at 21 week

LDR+HFD vs. HFD						LDR vs. Con					
Up			Down			Up			Down		
**Name**	**SC**	**FC**	**Name**	**SC**	**FC**	**Name**	**SC**	**FC**	**Name**	**SC**	**FC**
5-Methylcytidine	NNA	12.48103254	3-(Methylthio)-1-propene	OC	0.170974327	I-Urobilin	OC	18.12167485	Fenpropimorph	Benzenoids	0.112359454
Urothion	OC	10.13446036	LysoPC(16:1(9Z)/0:0)	LLM	0.223603688	Methylimidazoleacetic acid	OC	6.735013774	Uzarigenin 3-[xylosyl-(1->2)-rhamnoside]	LLM	0.1122379
Demethoxyshogaol	Benzenoids	5.766729979	beta-D-Glucosamine	OC	0.242509609	4-Hydroxy-2-butenoic acid gamma-lactone	OC	6.685993931	12-KETE	LLM	0.35897962
Falcarindiol	LLM	5.76436	1,2-Dihydroxy-3-keto-5-methylthiopentene	OOC	0.285668721	(-)-Epigallocatechin 3-cinnamate		4.968879712	Erinacine P	LLM	0.46371382
1,1'-[1,12-Dodecanediylbis(oxy)]bisbenzene	Benzenoids	4.912683299	Androstenedione	LLM	0.317655394	Dihydroprudomenin	PPP	4.471747626	3,5-Dimethylphenyl methylcarbamate	Benzenoids	0.479103954
Nutriacholic acid	LLM	4.786824122	Chalcone	PPP	0.329676007	Di-4-coumaroylputrescine	PPP	4.266030345	(Z)-2-Methyl-2-butene-1,4-diol 4-O-beta-D-Glucopyranoside	LLM	0.476545683
N-Acetylprocainamide	Benzenoids	4.77884637	LysoPE(16:1(9Z)/0:0)	LLM	0.340899303	Fagomine	OC	4.086452587	9,10-epoxyoctadecanoic acid	LLM	0.515589762
11-beta-Hydroxyandrosterone-3-glucuronide	LLM	2.546417796	5-(2-Hydroxyethyl)-4-methylthiazole	OC	0.354393739	6-Deoxyfagomine	OC	3.899104879			
LysoPC(24:1(15Z))	LLM	2.447781429	Thiamine	OC	0.350332488	Tyrosyl-Lysine	OAD	3.665641699			

Nandrolone	LLM	2.340743314	Isoleucyl-Alanine	OAD	0.371165388	Glycyl-Lysine	OAD	3.543330483
PE(16:0/18:2(9Z,12Z))	LLM	2.162521705	Asparaginyl-Leucine	OAD	0.377165124	N1,N10-Dicoumaroylspermidine	PPP	3.322625114
PE(14:0/15:0)	LLM	2.097706622	(Â±)-2-Pentanethiol	OC	0.382544262	3-Methylcytosine	OC	3.232675763
alpha-Methylphenylalanine	PPP	1.898378929	L-alpha-Aminobutyric acid	OAD	0.387339383	Cinnamyl benzoate	Benzenoids	3.146998869
pyrrolidinecarboxylic acid	OAD	1.898091289	Cortisone	LLM	0.399696008	11-beta-Hydroxyandrosterone-3-glucuronide	LLM	3.045193744
4-Guanidinobutanoic acid	OAD	1.877035957	Pyrimidine	OC	0.408848336	Linamarin	OOC	2.458520258
Phenylacetylglycine	OAD	1.643210714	3,4-Dihydro-6-methoxy-2,2-dimethyl-2H-1-benzopyran-4-ol	OC	0.43146372	4,4-Dimethylcholesta-8,14,24-trienol	LLM	2.321392806
3-beta-Hydroxy-4-beta-methyl-5-alpha-cholest-7-ene-4-alpha-carbaldehyde	LLM	1.595172367	Allixin	OC	0.444655748	Tryptophyl-Isoleucine	OAD	2.306253248
Cholesterol	LLM	1.586587003	L-Lysine	OAD	0.458439509	Yuccaol C	PPP	2.245793735
Valyl-Leucine	OAD	1.557129957	6,7-Dihydro-2,5-dimethyl-5H-cyclopentapyrazine	OC	0.46217978	Debrisoquine	OC	2.130815261
gamma-Aminobutyric acid	OAD	1.504957395	D-Alanine	OAD	0.461850144	6-Methylnicotinamide	OC	2.127845104

Metenamine	OC	1.267574166	4-(Methylnitrosamino)-1-(3-pyridyl)-1-butanol	OC	0.471237003	1-(Hydroxymethyl)-5,5-dimethyl-2,4-imidazolidinedione	OC	2.109546817

LLM: Lipids and lipid-like molecules; PPP: Phenylpropanoids and polyketides; OAD: Organic acids and derivatives; OOC: Organic oxygen compounds; ONC: Organic nitrogen compounds; NNA: Nucleosides, nucleotides, and analogues; OC: Organoheterocyclic compounds; FC: fold change; SC:superclass

Supplementary table 4 Histidine metabolism, phenylalanine metabolism, and the prolactinsignalling pathway

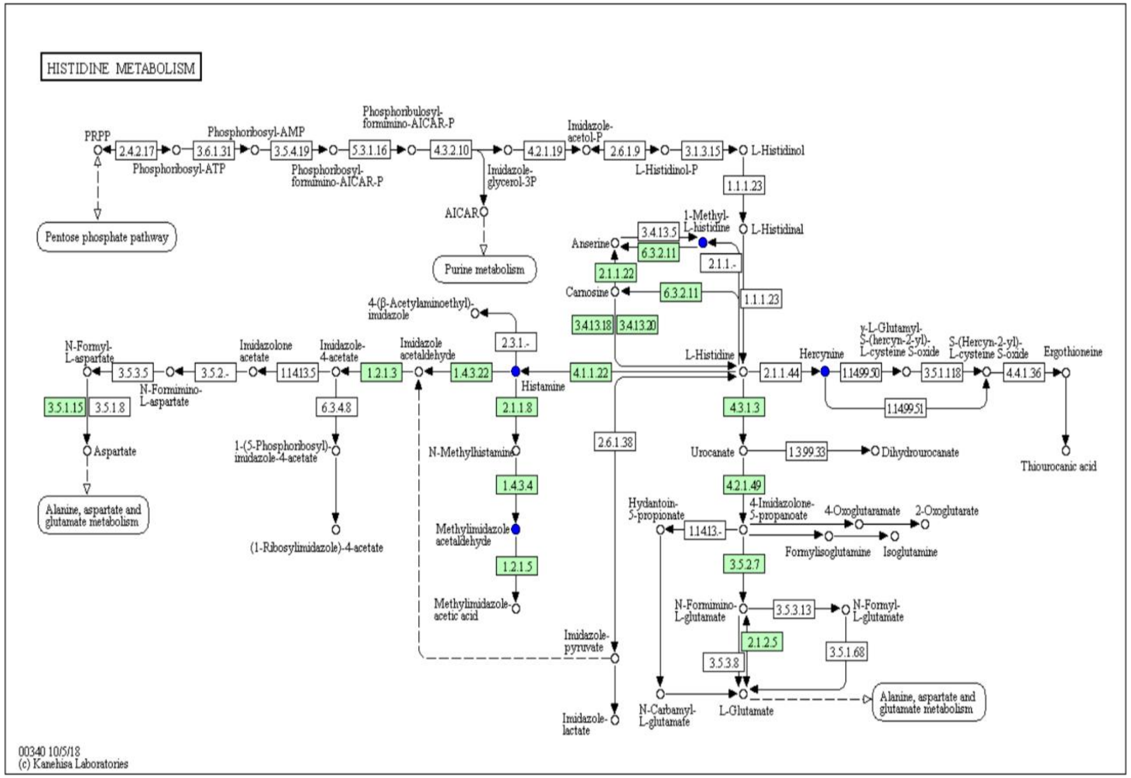
HISTIDINE METABOLISM
PRPP
2.4.2.17
Phosphoribosyl-ATP
3.6.1.31
Phosphoribosyl-AMP
3.5.4.19
Phosphoribosyl-formimino-AICAR-P
5.3.1.16
Phosphoribulosyl-formimino-AICAR-P
4.3.2.10
Imidazole-glycerol-3P
4.2.1.19
Imidazole-acetol-P
2.6.1.9
L-Histidinol-P
3.1.3.15
L-Histidinol
1.1.1.23
L-Histidinal
AICAR
Pentose phosphate pathway
Purine metabolism
Anserine
3.4.13.5
1-Methyl-L-histidine
6.3.2.11
2.1.1.-
2.1.1.22
Carnosine
3.4.13.18
3.4.13.20
4-(β-Acetylaminoethyl)-imidazole
2.3.1.-
N-Formyl-L-aspartate
3.5.3.5
N-Formimino-L-aspartate
3.5.2.-
Imidazolone acetate
1.14.13.5
Imidazole-4-acetate
1.2.1.3
Imidazole acetaldehyde
1.4.3.22
Histamine
4.1.1.22
L-Histidine
2.1.1.44
Hercynine
1.14.99.50
γ-L-Glutamyl-S-(hercyn-2-yl)-L-cysteine S-oxide
3.5.1.118
S-(Hercyn-2-yl)-L-cysteine S-oxide
4.4.1.36
Ergothioneine
1.14.99.51
Thiourocanic acid
3.5.1.15
3.5.1.8
Aspartate
6.3.4.8
1-(5-Phosphoribosyl)-imidazole-4-acetate
(1-Ribosylimidazole)-4-acetate
2.1.1.8
N-Methylhistamine
1.4.3.4
Methylimidazole acetaldehyde
1.2.1.5
Methylimidazole-acetic acid
2.6.1.38
Imidazole-pyruvate
Imidazole-lactate
4.3.1.3
Urocanate
1.3.99.33
Dihydrourocanate
4.2.1.49
Hydantoin-5-propionate
1.14.13.-
4-Imidazolone-5-propanoate
4-Oxoglutaramate
2-Oxoglutarate
Formylisoglutamine
Isoglutamine
3.5.2.7
N-Formimino-L-glutamate
3.5.3.13
N-Formyl-L-glutamate
2.1.2.5
3.5.3.8
3.5.1.68
N-Carbamyl-L-glutamate
L-Glutamate
Alanine, aspartate and glutamate metabolism
00340 10/5/18
(c) Kanehisa Laboratories

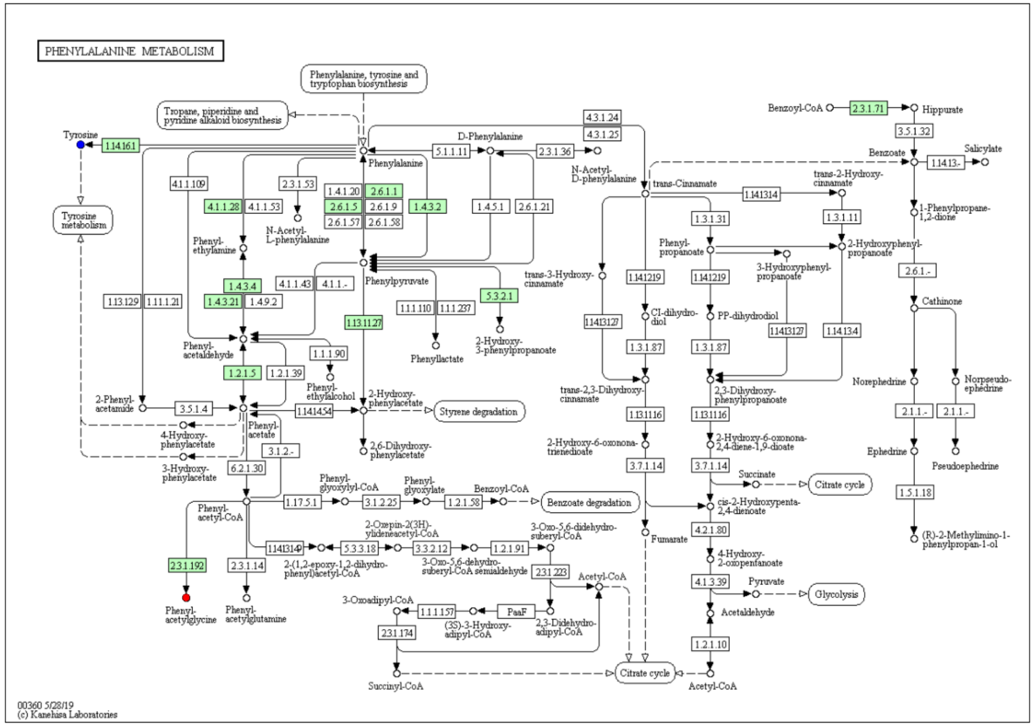

PHENYLALANINE METABOLISM
Phenylalanine, tyrosine and tryptophan biosynthesis
Tropane, piperidine and pyridine alkaloid biosynthesis
Tyrosine
1.14.16.1
Tyrosine metabolism
Phenylalanine
D-Phenylalanine
5.1.1.11
2.3.1.36
N-Acetyl-D-phenylalanine
4.3.1.24
4.3.1.25
4.1.1.109
4.1.1.28
4.1.1.53
2.3.1.53
N-Acetyl-L-phenylalanine
1.4.1.20
2.6.1.1
2.6.1.5
2.6.1.9
2.6.1.57
2.6.1.58
1.4.3.2
1.4.5.1
2.6.1.21
Phenylethylamine
Phenylpyruvate
1.4.3.4
1.4.3.21
1.4.9.2
4.1.1.43
4.1.1.-
1.13.12.9
1.11.21
1.13.11.27
1.1.1.110
1.1.1.237
5.3.2.1
trans-3-Hydroxycinnamate
2-Hydroxy-3-phenylpropanoate
Phenyllactate
Phenylacetaldehyde
1.1.1.90
Phenylethylalcohol
1.2.1.5
1.2.1.39
2-Phenylacetamide
3.5.1.4
1.14.14.54
2-Hydroxyphenylacetate
Styrene degradation
2,6-Dihydroxyphenylacetate
Phenylacetate
4-Hydroxyphenylacetate
3-Hydroxyphenylacetate
3.1.2.-
6.2.1.30
Phenylacetyl-CoA
1.17.5.1
Phenylglyoxylyl-CoA
3.1.2.25
Phenylglyoxylate
1.2.1.58
Benzoyl-CoA
Benzoate degradation
2.3.1.192
Phenylacetylglycine
2.3.1.14
Phenylacetylglutamine
1.14.13.149
2-(1,2-epoxy-1,2-dihydrophenyl)acetyl-CoA
5.3.3.18
2-Oxepin-2(3H)-ylideneacetyl-CoA
3.3.2.12
3-Oxo-5,6-dehydrosuberyl-CoA semialdehyde
1.2.1.91
3-Oxo-5,6-didehydrosuberyl-CoA
2.3.1.223
Acetyl-CoA
3-Oxoadipyl-CoA
1.1.1.157
(3S)-3-Hydroxyadipyl-CoA
PaaF
2,3-Didehydroadipyl-CoA
2.3.1.174
Succinyl-CoA
Citrate cycle
trans-Cinnamate
1.14.13.14
trans-2-Hydroxycinnamate
1.3.1.11
1.3.1.31
Phenylpropanoate
2-Hydroxyphenylpropanoate
3-Hydroxyphenylpropanoate
1.14.12.19
1.14.13.127
cis-3-(Carboxy-ethyl)-3,5-cyclohexadiene-1,2-diol
CI-dihydrodiol
PP-dihydrodiol
1.3.1.87
1.14.13.4
trans-2,3-Dihydroxycinnamate
2,3-Dihydroxyphenylpropanoate
1.13.11.16
2-Hydroxy-6-oxonona-trienedioate
2-Hydroxy-6-oxonona-2,4-diene-1,9-dioate
3.7.1.14
Succinate
Citrate cycle
cis-2-Hydroxypenta-2,4-dienoate
Fumarate
4.2.1.80
4-Hydroxy-2-oxopentanoate
4.1.3.39
Pyruvate
Glycolysis
Acetaldehyde
1.2.1.10
Acetyl-CoA
Benzoyl-CoA
2.3.1.71
Hippurate
3.5.1.32
Benzoate
1.14.13.-
Salicylate
1-Phenylpropane-1,2-dione
2.6.1.-
Cathinone
Norephedrine
Norpseudoephedrine
2.1.1.-
Ephedrine
Pseudoephedrine
1.5.1.18
(R)-2-Methylimino-1-phenylpropan-1-ol
00360 5/28/19
(c) Kanehisa Laboratories

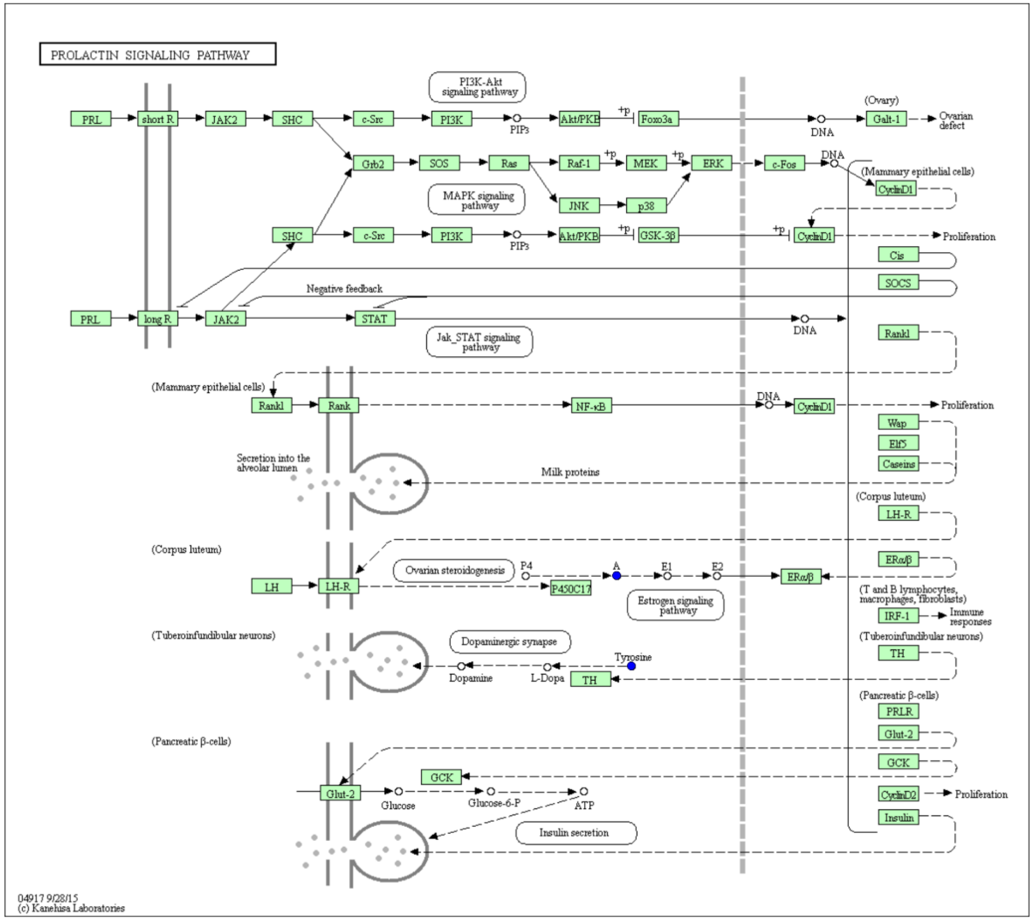

Supplementary Table5 Pathway of sphingolipid metabolism, proline metabolism, and glycerophospholipid metabolism

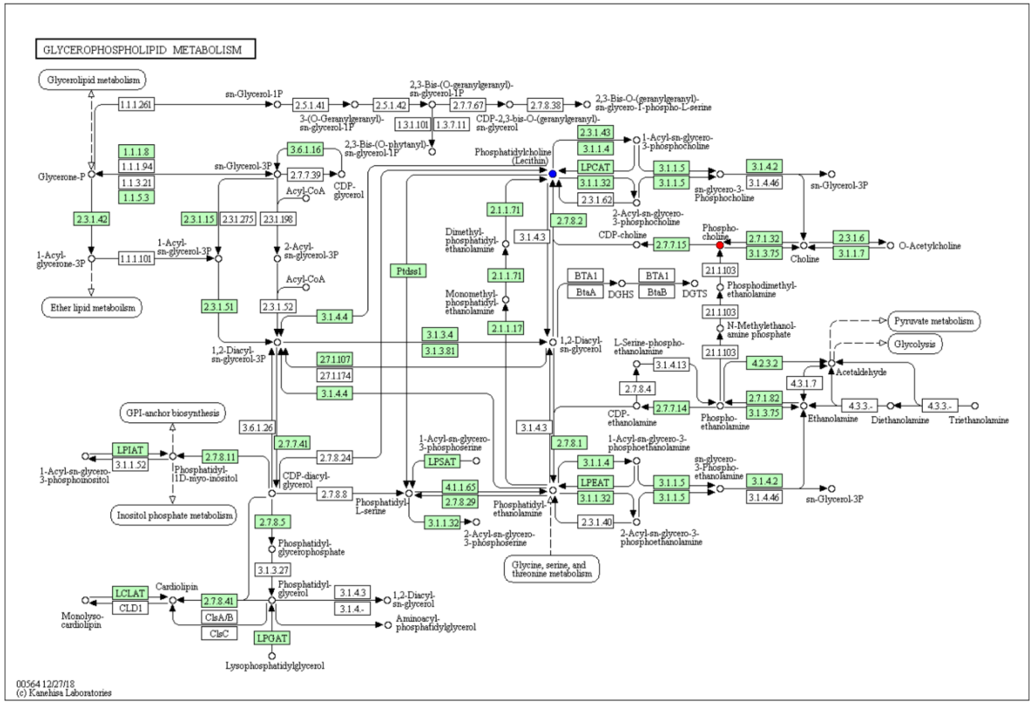
GLYCEROPHOSPHOLIPID METABOLISM
Glycerolipid metabolism
sn-Glycerol-1P
2,3-Bis-(O-geranylgeranyl)-sn-glycerol-1P
2,3-Bis-O-(geranylgeranyl)-sn-glycero-1-phospho-L-serine
3-(O-Geranylgeranyl)-sn-glycerol-1P
CDP-2,3-bis-O-(geranylgeranyl)-sn-glycerol
2,3-Bis-(O-phytanyl)-sn-glycerol-1P
Glycerone-P
sn-Glycerol-3P
Acyl-CoA
CDP-glycerol
1-Acyl-glycerone-3P
1-Acyl-sn-glycerol-3P
2-Acyl-sn-glycerol-3P
Ether lipid metabolism
1,2-Diacyl-sn-glycerol-3P
Phosphatidylcholine (Lecithin)
1-Acyl-sn-glycero-3-phosphocholine
sn-glycero-3-Phosphocholine
2-Acyl-sn-glycero-3-phosphocholine
CDP-choline
Phospho-choline
Choline
O-Acetylcholine
Dimethyl-phosphatidyl-ethanolamine
Monomethyl-phosphatidyl-ethanolamine
DGHS
DGTS
Phosphodimethyl-ethanolamine
N-Methylethanol-amine phosphate
1,2-Diacyl-sn-glycerol
L-Serine-phospho-ethanolamine
Pyruvate metabolism
Glycolysis
Acetaldehyde
Ethanolamine
Diethanolamine
Triethanolamine
CDP-ethanolamine
Phospho-ethanolamine
GPI-anchor biosynthesis
1-Acyl-sn-glycero-3-phosphoinositol
Phosphatidyl-1D-myo-inositol
Inositol phosphate metabolism
CDP-diacyl-glycerol
1-Acyl-sn-glycero-3-phosphoserine
Phosphatidyl-L-serine
Phosphatidyl-ethanolamine
1-Acyl-sn-glycero-3-phosphoethanolamine
sn-glycero-3-Phospho-ethanolamine
2-Acyl-sn-glycero-3-phosphoserine
2-Acyl-sn-glycero-3-phosphoethanolamine
Glycine, serine, and threonine metabolism
Phosphatidyl-glycerophosphate
Phosphatidyl-glycerol
Cardiolipin
Monolyso-cardiolipin
1,2-Diacyl-sn-glycerol
Aminoacyl-phosphatidylglycerol
Lysophosphatidylglycerol
1.1.1.261
2.5.1.41
2.5.1.42
2.7.7.67
2.7.8.38
1.3.1.101
1.3.7.11
1.1.1.8
1.1.1.94
1.1.3.21
1.1.5.3
3.6.1.16
2.7.7.39
2.3.1.42
2.3.1.15
2.3.1.275
2.3.1.198
1.1.1.101
2.3.1.51
2.3.1.52
Ptdss1
2.3.1.43
3.1.1.4
LPCAT
3.1.1.32
2.3.1.62
3.1.1.5
3.1.4.2
3.1.4.46
2.1.1.71
2.1.1.17
2.7.8.2
3.1.4.3
2.7.7.15
2.7.1.32
3.1.3.75
2.3.1.6
3.1.1.7
BTA1
BtaA
BtaB
2.1.1.103
3.1.4.4
3.1.3.4
3.1.3.81
2.7.1.107
2.7.1.174
3.1.4.13
2.7.8.4
4.2.3.2
4.3.1.7
2.7.1.82
2.7.7.14
4.3.3.-
3.6.1.26
2.7.7.41
2.7.8.24
LPIAT
3.1.1.52
2.7.8.11
LPSAT
2.7.8.1
LPEAT
2.3.1.40
2.7.8.8
4.1.1.65
2.7.8.29
2.7.8.5
3.1.3.27
LCLAT
CLD1
2.7.8.41
ClsA/B
ClsC
LPGAT
3.1.4.-
00564 12/27/18
(c) Kanehisa Laboratories

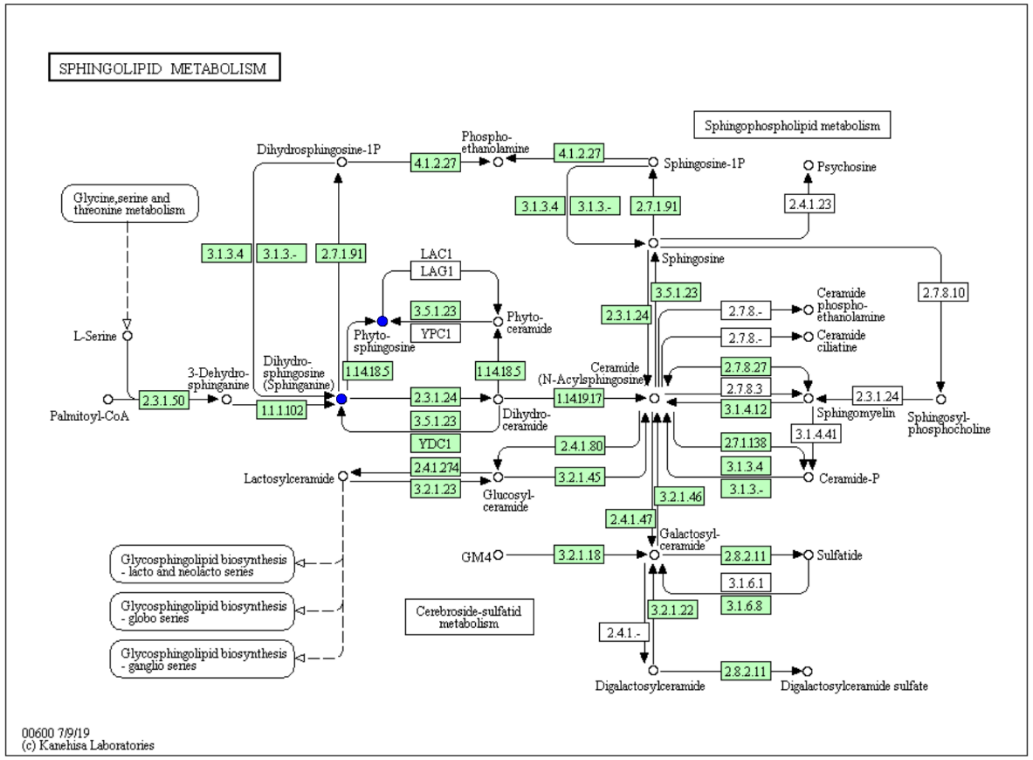
SPHINGOLIPID METABOLISM
Sphingophospholipid metabolism
Dihydrosphingosine-1P
4.1.2.27
Phospho-
ethanolamine
4.1.2.27
Sphingosine-1P
Psychosine
Glycine,serine and
threonine metabolism
3.1.3.4
3.1.3.-
2.7.1.91
2.4.1.23
3.1.3.4
3.1.3.-
2.7.1.91
LAC1
LAG1
Sphingosine
2.7.8.10
3.5.1.23
3.5.1.23
Phyto-
sphingosine
YPC1
Phyto-
ceramide
2.3.1.24
2.7.8.-
Ceramide
phospho-
ethanolamine
2.7.8.-
Ceramide
ciliatine
L-Serine
1.14.18.5
1.14.18.5
3-Dehydro-
sphinganine
Dihydro-
sphingosine
(Sphinganine)
Ceramide
(N-Acylsphingosine)
2.7.8.27
2.7.8.3
3.1.4.12
Palmitoyl-CoA
2.3.1.50
1.1.1.102
2.3.1.24
3.5.1.23
YDC1
Dihydro-
ceramide
1.14.19.17
2.3.1.24
Sphingomyelin
Sphingosyl-
phosphocholine
3.1.4.41
2.4.1.80
2.7.1.138
3.1.3.4
3.1.3.-
Ceramide-P
Lactosylceramide
2.4.1.274
3.2.1.23
Glucosyl-
ceramide
3.2.1.45
3.2.1.46
2.4.1.47
Galactosyl-
ceramide
GM4
3.2.1.18
2.8.2.11
Sulfatide
3.1.6.1
3.1.6.8
3.2.1.22
2.4.1.-
Glycosphingolipid biosynthesis
- lacto and neolacto series
Glycosphingolipid biosynthesis
- globo series
Glycosphingolipid biosynthesis
- ganglio series
Cerebroside-sulfatid
metabolism
Digalactosylceramide
2.8.2.11
Digalactosylceramide sulfate
00600 7/9/19
(c) Kanehisa Laboratories

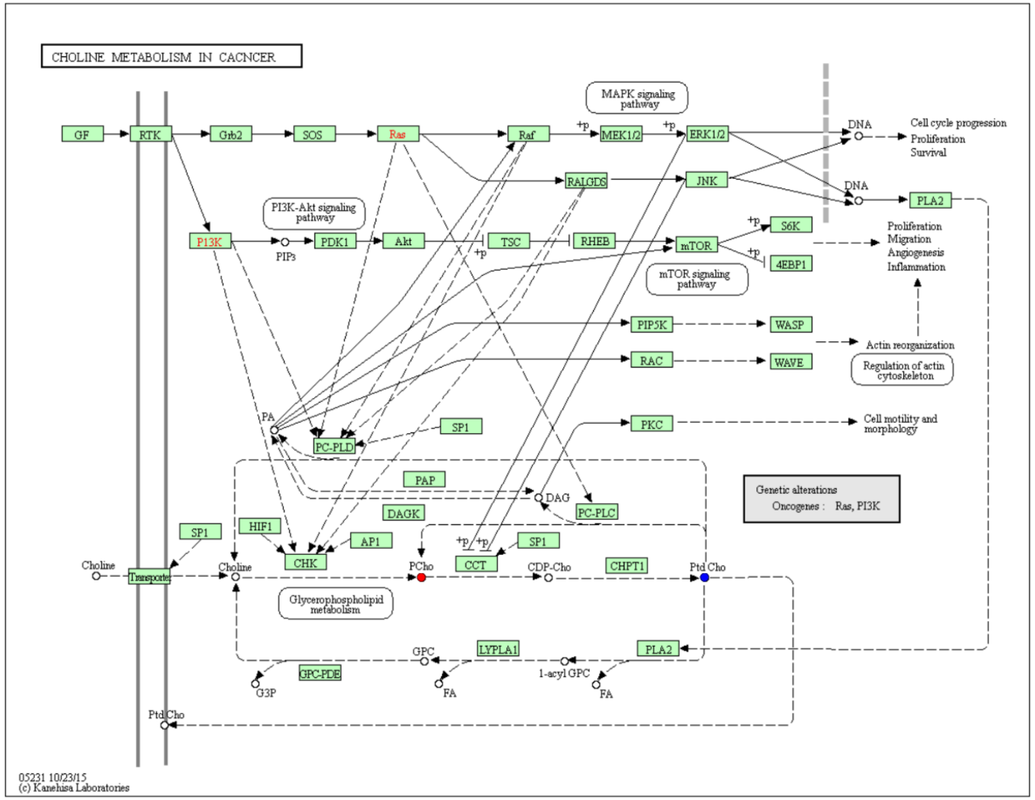
CHOLINE METABOLISM IN CACNCER
MAPK signaling pathway
GF
RTK
Grb2
SOS
Ras
Raf
MEK1/2
ERK1/2
DNA
Cell cycle progression
Proliferation
Survival
RALGDS
JNK
DNA
PLA2
PI3K-Akt signaling pathway
PI3K
PIP3
PDK1
Akt
TSC
RHEB
mTOR
S6K
4EBP1
Proliferation
Migration
Angiogenesis
Inflammation
mTOR signaling pathway
PIP5K
WASP
Actin reorganization
RAC
WAVE
Regulation of actin cytoskeleton
PA
SP1
PC-PLD
PKC
Cell motility and morphology
PAP
DAG
DAGK
PC-PLC
Genetic alterations
Oncogenes : Ras, PI3K
SP1
HIF1
AP1
SP1
Choline
Transporter
Choline
CHK
PCho
CCT
CDP-Cho
CHPT1
Ptd Cho
Glycerophospholipid metabolism
GPC
LYPLA1
1-acyl GPC
PLA2
G3P
GPC-PDE
FA
FA
Ptd Cho
05231 10/23/15
(c) Kanehisa Laboratories
