# Supplementary material for: Low-dose radiation exaggerates HFD-induced metabolic dysfunction by gut microbiota through PA-PYCR1 axis
Source: Commun Biol. 2022 Sep 10;5:945. doi: 10.1038/s42003-022-03929-1 (PMC9464247; doi:10.1038/s42003-022-03929-1)
Supplement: Supplementary file 4 — Reporting Summary [file 42003_2022_3929_MOESM4_ESM.pdf]

Corresponding author(s): Ruixue Huang

Last updated by author(s): Apr 29, 2022

Reporting Summary

Nature Research wishes to improve the reproducibility of the work that we publish. This form provides structure for consistency and transparency in reporting. For further information on Nature Research policies, see our Editorial Policies and the Editorial Policy Checklist.

Statistics

For all statistical analyses, confirm that the following items are present in the figure legend, table legend, main text, or Methods section.

n/a	Confirmed	
☐	☒	The exact sample size (*n*) for each experimental group/condition, given as a discrete number and unit of measurement
☐	☒	A statement on whether measurements were taken from distinct samples or whether the same sample was measured repeatedly
☒	☐	The statistical test(s) used AND whether they are one- or two-sided<br>*Only common tests should be described solely by name; describe more complex techniques in the Methods section.*
☒	☐	A description of all covariates tested
☒	☐	A description of any assumptions or corrections, such as tests of normality and adjustment for multiple comparisons
☐	☒	A full description of the statistical parameters including central tendency (e.g. means) or other basic estimates (e.g. regression coefficient) AND variation (e.g. standard deviation) or associated estimates of uncertainty (e.g. confidence intervals)
☒	☐	For null hypothesis testing, the test statistic (e.g. *F*, *t*, *r*) with confidence intervals, effect sizes, degrees of freedom and *P* value noted<br>*Give P values as exact values whenever suitable.*
☒	☐	For Bayesian analysis, information on the choice of priors and Markov chain Monte Carlo settings
☐	☒	For hierarchical and complex designs, identification of the appropriate level for tests and full reporting of outcomes
☒	☐	Estimates of effect sizes (e.g. Cohen's *d*, Pearson's *r*), indicating how they were calculated

Our web collection on statistics for biologists contains articles on many of the points above.

Software and code

Policy information about availability of computer code

Data collection	N/A
Data analysis	N/A

For manuscripts utilizing custom algorithms or software that are central to the research but not yet described in published literature, software must be made available to editors and reviewers. We strongly encourage code deposition in a community repository (e.g. GitHub). See the Nature Research guidelines for submitting code & software for further information.

Data

Policy information about availability of data

All manuscripts must include a data availability statement. This statement should provide the following information, where applicable:

- Accession codes, unique identifiers, or web links for publicly available datasets
- A list of figures that have associated raw data
- A description of any restrictions on data availability

Data are applicable under the request to correspondence author.

Field-specific reporting

Please select the one below that is the best fit for your research. If you are not sure, read the appropriate sections before making your selection.

☒ Life sciences ☐ Behavioural & social sciences ☐ Ecological, evolutionary & environmental sciences

For a reference copy of the document with all sections, see nature.com/documents/nr-reporting-summary-flat.pdf

Life sciences study design

All studies must disclose on these points even when the disclosure is negative.

Sample size	No statisctial methods were used to predetermine sample sizes. we ensured that they were similar to those generally employed in the field
Data exclusions	Samples with no detectable related to fig1-8 were excluded. no other exclusions were made.
Replication	To ensure the reproducibility of the experimental finding , we have used 3-4 individual mice for each sample group. all attempts at replication were successfully done.
Randomization	For all experiments, mice used were randomly allocated into each experimental group by our team.
Blinding	Blinding was not possible because we needed to know how the parameters results from each group of mice during sample collection and for analysis.

Reporting for specific materials, systems and methods

We require information from authors about some types of materials, experimental systems and methods used in many studies. Here, indicate whether each material, system or method listed is relevant to your study. If you are not sure if a list item applies to your research, read the appropriate section before selecting a response.

Materials & experimental systems

n/a	Involved in the study	
☐	☒	Antibodies
☒	☐	Eukaryotic cell lines
☒	☐	Palaeontology and archaeology
☐	☒	Animals and other organisms
☒	☐	Human research participants
☒	☐	Clinical data
☒	☐	Dual use research of concern

Methods

n/a	Involved in the study	
☒	☐	ChIP-seq
☒	☐	Flow cytometry
☒	☐	MRI-based neuroimaging

Antibodies

Antibodies used	The antibodies used for Western blot analysis targeted the following proteins: GAPDH(CST, 5174), E-cadherin(CST, 14472), PI3K(Abcam, ab73262), Akt (Proteintech, 10176-2-AP), p-Akt (Abcam, ab81283), IRS-1 (CST, 2382), p-IRS-1 (CST, 2381), mTOR (Bioworld, bs1844), p-mTOR (CST, 5536S), PYCR1 (Abcam, ab279385), p53 (CST, 2527S), p-PI3K(CST, 4228T), Occludin(Abcam, ab216327), ZO-1 (Abcam, ab221547), Claudin-1 (Abcam, ab211737), CLDN (Abcam, ab207300), N-cadherin(CST, 31593), IFN-γ (Abcam, ab224197), MLCK(Abcam, ab76092), IL-17A (Abcam, ab79056), p-ERK(Abcam, ab279084), GSK3β (Abcam, ab32391), INSR (CST, 23413), BAD (Abcam, ab32445), and TC10 (Abcam, ab32079). An antibiotic cocktail including ampicillin sodium salt (CA2031-5G), polymyxin B sulfate salt (CP8711-100MG) and streptomycin sulfate (CS 10481-5G) was purchased from Coolaber Science & Technology. The probiotics includedLactobacillus rhamnosus(No. CICC 20259) and Lactobacillus reuteri (No. CICC 6118), which were purchased from the China Center of Industrial Culture Collection (http://www.china-cicc.org/).PA was purchased from Solarbio (p0011). Insulin was purchased from Coolaber Science & Technology (CI6561), China.
Validation	All antibodies used were validated.

Animals and other organisms

Policy information about studies involving animals; ARRIVE guidelines recommended for reporting animal research

Laboratory animals	Male C57BL/6 mice were purchased at 8 weeks of age from the Fengtai Animal Center, Beijing, China.
Wild animals	N/A

Field-collected samples	N/A
Ethics oversight	The study and research protocols were approved by the Institutional Animal Review Board of Central South University (2020sydw0110).

Note that full information on the approval of the study protocol must also be provided in the manuscript.
